# Experiences of Using Digital Mindfulness-Based Interventions: Rapid Scoping Review and Thematic Synthesis

**DOI:** 10.2196/44220

**Published:** 2023-09-28

**Authors:** Emma Louise Osborne, Ben Ainsworth, Nic Hooper, Melissa Jayne Atkinson

**Affiliations:** 1 Department of Psychology University of Bath Bath United Kingdom; 2 School of Psychology University of Southampton Southampton United Kingdom; 3 School of Psychology Cardiff University Cardiff United Kingdom

**Keywords:** mindfulness, digital intervention, dropout, eHealth, engagement, mobile health, mHealth, psychosocial intervention, qualitative research, scoping review, thematic synthesis, mobile phone

## Abstract

**Background:**

Digital mindfulness-based interventions (MBIs) are a promising approach to deliver accessible and scalable mindfulness training and have been shown to improve a range of health outcomes. However, the success of digital MBIs is reliant on adequate engagement, which remains a crucial challenge. Understanding people’s experiences of using digital MBIs and identifying the core factors that facilitate or act as barriers to engagement is essential to inform intervention development and maximize engagement and outcomes.

**Objective:**

This study aims to systematically map the literature on people’s experiences of using digital MBIs that target psychosocial variables (eg, anxiety, depression, distress, and well-being) and identify key barriers to and facilitators of engagement.

**Methods:**

We conducted a scoping review to synthesize empirical qualitative research on people’s experiences of using digital MBIs. We adopted a streamlined approach to ensure that the evidence could be incorporated into the early stages of intervention development. The search strategy identified articles with at least one keyword related to mindfulness, digital, user experience, and psychosocial variables in their title or abstract. Inclusion criteria specified that articles must have a qualitative component, report on participants’ experiences of using a digital MBI designed to improve psychosocial variables, and have a sample age range that at least partially overlapped with 16 to 35 years. Qualitative data on user experience were charted and analyzed using inductive thematic synthesis to generate understandings that go beyond the content of the original studies. We used the Quality of Reporting Tool to critically appraise the included sources of evidence.

**Results:**

The search identified 530 studies, 22 (4.2%) of which met the inclusion criteria. Overall, the samples were approximately 78% female and 79% White; participants were aged between 16 and 69 years; and the most used measures in intervention studies were mindfulness, psychological flexibility, and variables related to mental health (including depression, anxiety, stress, and well-being). All studies were judged to be adequately reported. We identified 3 themes characterizing barriers to and facilitators of engagement: *responses to own practice* (ie, negative reactions to one’s own practice are common and can deplete motivation), *making mindfulness a habit* (ie, creating a consistent training routine is essential yet challenging), and *leaning on others* (ie, those engaging depend on someone else for support).

**Conclusions:**

The themes identified in this review provide crucial insights as to why people frequently stop engaging with digital MBIs. Researchers and developers should consider using person-based coparticipatory methods to improve acceptability of and engagement with digital MBIs, increase their effectiveness, and support their translation to real-world use. Such strategies must be grounded in relevant literature and meet the priorities and needs of the individuals who will use the interventions.

## Introduction

### Background

Mindfulness involves (1) attentional monitoring of present-moment experience (eg, thoughts, feelings, and sensations) and (2) orientation toward this experience with acceptance and nonjudgment [1]. Mindfulness-based interventions (MBIs) aim to train these skills and have been shown to improve a range of psychological and physical health outcomes in both clinical and nonclinical populations. For example, there is evidence from meta-analyses of randomized controlled trials suggesting that MBIs can reduce depression and anxiety or stress in young people [2], lower pain intensity in patients with chronic pain [3], and reduce symptoms of posttraumatic stress in people with and without a diagnosis [4].

Despite such efficacy, there are numerous challenges in accessing and delivering MBIs, including geographical, logistical, and financial constraints as well as a lack of trained mindfulness teachers [5,6]. For example, MBIs are typically face-to-face, multisession, and facilitated by expert interventionists, such as the mindfulness-based cognitive therapy (MBCT) course that is traditionally delivered by dedicated instructors in 8 weekly 2-hour group training sessions [7]. The translation of MBIs into digital formats has the potential to overcome these constraints, and it is encouraging that early evaluations of digital MBIs report beneficial effects that are comparable with those found in traditional in-person programs [8,9].

However, unfortunately, the success of digital MBIs is reliant on adequate engagement, which remains a crucial challenge. Engagement refers to the investment of energy in an activity and includes physical (ie, actual performance, which researchers often rely on when examining engagement using objective behavioral metrics [10]), affective (ie, affective reactions), and cognitive (ie, selective attention) elements [11]. For example, reviews of digital MBIs have found that between 8% and 52% to 60% of participants do not complete all sessions [9,12]. Although low engagement is a common issue in digital mental health interventions generally [13]—for example, the pooled completion rate from studies of apps for depressive symptoms is 52% [14]—it is particularly important in mindfulness training as regular practice is essential to develop mindfulness skills. Time spent practicing mindfulness at home is related to increases in levels of mindfulness and, in turn, improvements in psychological functioning [15]. Similarly, those who report high levels of engagement with digital MBIs report greater improvement in outcomes than those who do not [12].

Given that the success of digital MBIs is related to engagement and engagement tends to be low with digital MBIs, understanding the factors that facilitate or act as barriers to engagement with these interventions is crucial to promote engagement and opportunities to benefit. Past research has suggested that there is a range of factors that influence adherence to digital MBIs [5], including accessibility (eg, across devices and populations with different needs), tailoring (eg, of content to individual needs), and difficulty (eg, sustaining attention). In one study, after engaging with a digital MBI, students with no meditation experience reported that the top 3 obstacles to practice from a checklist of common challenges were meditation feeling like “just another task,” “feeling distracted,” and “feeling sleepy” [16]. However, the use of closed-response questions in such research potentially prohibits the development of a detailed understanding that is grounded in people’s own perspectives regarding aspects that help them engage and hinder them from engaging [17].

A more detailed approach using inductive qualitative analysis examined factors that hindered or facilitated the engagement of 16 health care professionals who participated in a self-help MBI (participants could choose a printed book or a web-based program) [18]. The results indicated that longer practices, arising negative thoughts, and self-criticism were key hindrances, and shorter practices, motivation to reduce stress, and feelings of control over thoughts were key facilitators. However, over half of the participants opted for the book-based intervention in this study, and themes identified from engaging with the web-based and book-based MBIs were combined. Although the authors reported that themes were comparable across intervention types, it is possible that barriers and facilitators specific to the web-based version were obscured by those common to both. Therefore, it is unclear whether these themes would apply to typical digital MBIs as well as to other populations (eg, groups who are vulnerable to or experiencing clinical-level concerns or for whom initial engagement is lower).

Although some studies have reported on factors that can influence engagement with digital MBIs, they rarely build a deep understanding of users’ experiences or do so systematically. User-centered design approaches (such as the person-based approach [19]) emphasize that understanding how people use digital MBIs and identifying core barriers to and facilitators of engagement are important first steps in intervention development, which suggest key design objectives to ensure interventions are relevant, acceptable, and engaging to target users before significant investment is made in evaluation and implementation [20]. This is particularly important in the context of digital *mindfulness* interventions as, unlike most digital health interventions, engagement with the digital content is designed to facilitate completion of a concurrent nondigital target behavior that is metacognitive in nature (eg, an experiential mindfulness exercise) [11]. As factors influencing engagement vary across different target behaviors, clear guidance is needed to understand which are directly relevant to and most prominent in digital MBIs specifically.

### Objectives

This review aimed to synthesize qualitative evidence on individuals’ experiences of using digital MBIs targeting psychosocial variables (eg, anxiety, depression, distress, and well-being) to identify key barriers to and facilitators of engagement. We chose to perform a rapid scoping review of qualitative data as (1) factors influencing the effects of interventions are often rooted in variations in attitudes, opinions, thoughts, feelings, and behaviors and, therefore, best explored through qualitative study [21]; (2) qualitative evidence is necessary to understand engagement in its entirety (ie, its physical, cognitive, and affective components [11]); and (3) it ensures that existing evidence can be incorporated into the early stages of intervention development and implementation [22,23]. The knowledge generated from this review will inform the evaluation and development of new and existing digital MBIs, helping them overcome some of the challenges that individuals face when engaging with these interventions.

## Methods

### Overview

We adhere to the Enhancing Transparency in Reporting the Synthesis of Qualitative Research guidelines [24] in reporting this review, and the review itself was guided by the Cochrane Rapid Reviews Methods recommendations [25] and PRISMA-ScR (Preferred Reporting Items for Systematic Reviews and Meta-Analyses Extension for Scoping Reviews; Multimedia Appendix 1 [26]). We developed and preregistered an a priori protocol that specified the review questions (What are the key barriers to and facilitators of engagement with digital MBIs targeting psychosocial variables? How have interventions addressed and used these barriers and facilitators in the past, and in what ways could interventions address and use them in future?); participants, intervention, comparison, outcome, and study design; electronic database; search strategy; inclusion and exclusion criteria; and data charting form [27].

### Inclusion and Exclusion Criteria

The inclusion and exclusion criteria were developed to identify qualitative explorations of individuals’ perspectives and experiences of using digital MBIs designed to improve psychosocial variables (Textbox 1). We excluded studies that did not refer to a digital web-based intervention (eg, a biofeedback headband and device based on vapor, light, and sound, both designed to support mindful breathing) and studies of interventions in which mindfulness was not the main component (eg, an intervention composed of 3 evidence-based techniques: cognitive behavioral coaching, motivational interviewing, and mindfulness). We specified that sample age ranges must at least partially overlap with 16 to 35 years as this is the target age group for our own intervention development. We defined *digital* MBIs as those delivered via the web by the technology itself (eg, hardware and electronic devices, software, and websites) rather than by health care professionals remotely [28]. Human support (eg, answering questions; providing feedback; and offering coaching, orientation, or check-in sessions) was permitted where the support was considered supplementary to the delivery of content, and we reported on the presence and format of such support in each included study. We focused on peer-reviewed papers as they would have received some initial quality assessment. Nonreporting bias [29] was minimized in this review as its focus was on generating themes related to engagement rather than estimating effects (ie, we did not extract quantitative results and included studies with no reported quantitative outcomes).

**Inclusion and exclusion criteria for the selected articles.**

**Inclusion criteria**

- Type of publication: peer-reviewed empirical article (ie, original research based on observation or experiment)
- Language: published in English
- Study design: qualitative or mixed methods study or an intervention study with a qualitative component (including free text from questionnaire surveys); may report on a full-scale or pilot-scale project
- Phenomena of interest: any information on experiences of using a digital web-based mindfulness-based intervention (an intervention—research or commercially available—in which mindfulness is the main component) designed to improve psychosocial variables (ie, not interventions that solely target physiological variables); if an intervention study, must use psychosocial outcome or process measures
- Participants: sample age range at least partially overlapping with 16-35 years

**Exclusion criteria**

- Type of publication: not peer-reviewed or a review article (ie, does not contain original research)
- Language: not published in English
- Study design: does not include a qualitative component (including free text from questionnaire surveys)
- Phenomena of interest: does not include any information on experiences of using a digital web-based mindfulness-based intervention (an intervention in which mindfulness is the main component) or is an intervention study that does not use psychosocial outcome or process measures
- Participants: sample age range is entirely <16 years and/or >35 years

### Search Strategy

In consultation with an information specialist (psychology librarian who has extensive training in implementing structured database searches), we developed a comprehensive search strategy to identify articles with at least one keyword related to mindfulness, digital, user experience, and psychosocial variables in its title or abstract (Textbox 2). Keywords for psychosocial variables were derived from models of disordered eating [30] (ie, specific focus for our own intervention development), with added terms to broaden the search for all psychosocial variables (eg, affect, mood, distress, and well-being).

**Keywords (in the title or abstract) used during the search.**

**Search strategy**

- mindfu* AND internet OR online OR digital OR web OR e-health OR ehealth OR telemonit* OR computer* OR technolog* OR telecommunication* OR “tele communication*” OR multimedia OR pc OR website OR www OR “cell* phone” OR mobile OR smartphone OR “smart phone” OR electronic OR mhealth OR m-health OR telemedicine OR “tele medicine” OR “text messag*” OR email* OR telehealth OR “tele health” OR teletherap* OR “tele therap*” AND qualitative OR interview* OR “focus group*” OR experience* OR view* OR perspective* OR feedback OR ethnograp* OR “ethno grap*” OR thematic OR theme* OR “mixed methods” OR mixedmethod* OR “mixed method*” OR usability OR acceptab* OR feasib* OR thinkaloud OR “think aloud” OR open-ended OR semi-structured OR person-based OR “user cent*” OR participatory OR “human cent*” AND anxiet* OR depressi* OR affect* OR dysphori* OR mood OR emotion* OR distress OR wellbeing OR well-being OR negative OR “permissive thoughts” OR “maladaptive cognitions” OR “cognitive rigidity” OR interoceptive OR intero-ceptive OR acceptance OR self-esteem OR body* OR weight OR shape OR appearance OR eating OR diet* OR thin OR pressure* OR media OR perfectio* OR ineffectiveness OR self-efficacy OR selfefficacy OR self-concept OR selfconcept OR self-awareness OR selfawareness OR interpersonal OR inter-personal

### Screening

We uploaded the search results to Covidence (Veritas Health Innovation), a web-based systematic review software, to streamline the screening process. Consistent with guidance from the Agency for Healthcare Research and Quality [31], we started with a pilot phase to calibrate and test the eligibility criteria. In total, 2 researchers independently screened a random selection of 50 studies (10% of the records) and then met to resolve discrepancies (Multimedia Appendix 2 [32]). The first author screened the remaining titles and abstracts. All potentially eligible records were obtained as full-text articles. We requested full texts via our institution’s interlibrary loan service if they were unavailable on the web. The first author screened the full texts for inclusion in consultation with the wider research team, and the research team verified the final list of included articles.

### Data Charting

We used a pilot-tested form to record study characteristics and qualitative data on user experience (Multimedia Appendix 3). In total, 2 researchers independently charted data from a full text using a template adapted from the example evidence table for qualitative studies developed by the National Institute for Health and Care Excellence [33] and then met to discuss inconsistencies and improvements (Multimedia Appendix 4 [33]). The first author charted the remaining data. Our inclusive approach included qualitative data from any study type, such as qualitative data from qualitative studies (ie, studies that used a qualitative method of data collection *and* analysis), narrative data from qualitative components of mixed methods studies, and free text from questionnaire surveys as various types of qualitative evidence can enrich a synthesis [23]. In this study, charted qualitative data included quotations from participants and themes, theories, and interpretations generated by the studies’ authors. They were presented as narratives or summarized in tables and located in the *Abstract*, *Results*, and *Discussion* sections. We charted all qualitative data related to user experience as verbatim quotations. Multimedia Appendix 5 [34,35] provides a 17-page excerpt from our extensive data charting table.

### Critical Appraisal

We used the Quality of Reporting Tool [36] to critically appraise the included sources of evidence. The reporting of each study was appraised using 4 criteria: study design and research question, participant selection, data collection, and analysis. We assessed all qualitative studies overall (ie, as a whole) and all remaining papers (ie, mixed methods studies or questionnaire surveys) both overall and considering only qualitative data on user experience (ie, data included in our qualitative evidence synthesis). After pilot-testing the tool with 2 reviewers, a single reviewer categorized studies as “adequately reported” (satisfied at least 2 criteria) or “inadequately reported” (satisfied 1 or no criteria), and the first author verified all judgments and supporting evidence. These criteria have been used in other validated tools (eg, they represent items 3, 4, 5, and 8 from the Critical Appraisal Skills Programme qualitative checklist [37]) and in a review of barriers to and facilitators of engagement with digital mental health interventions [13].

### Data Analysis

As recommended in the Cochrane Handbook for Systematic Reviews of Interventions [23], we thematically synthesized charted qualitative data [38]. Thematic synthesis offers a clear and accessible inductive approach to produce descriptive themes that can evolve beyond the content of the primary studies into more in-depth analytic themes. The first author imported all charted qualitative data verbatim into the NVivo qualitative data analysis software (QSR International) and freely coded the data line by line according to their meaning and content using words directly from the data where possible. As qualitative evidence syntheses have received criticism for decontextualizing the findings of individual studies [38], the first author read all the charted data (including study aims, methods, and samples) before coding each study’s findings to preserve its original context and ensure that its findings could be fully understood without misinterpretation [39]. The first author then grouped similar codes into “descriptive themes” to summarize their meaning while keeping close to the original findings of the included studies. This was an iterative process that distilled users’ perspectives and experiences of using digital MBIs down to their key parts. In the next stage, the wider research team met to discuss the descriptive themes and develop “analytical themes,” which go beyond the findings of the primary studies by interpreting the key messages underlying the descriptive themes and using them to answer the review questions. We generated more abstract and analytical themes through an iterative process of inferring barriers, facilitators, and implications for intervention development from the descriptive themes and making changes to them where necessary. Multimedia Appendix 6 [23,38,39] provides more details about the analysis, including a 4-page excerpt from our list of codes, a full list of descriptive themes, and a comprehensive example of how we generated the analytical themes.

### Methodological Streamlining

We took several steps to accelerate the review process so that evidence could be quickly incorporated into the initial phase of intervention planning [40]. First, we limited the inclusion criteria to English-language publications [25]. Second, we restricted the search to PsycINFO as an efficient way to achieve a manageable amount of relevant data (ie, by using a specialist database for psychological interventions [41] to retrieve studies most suitable for answering our review questions). This was necessary given that (1) too much data because of a large number of included studies can undermine qualitative evidence syntheses and (2) other methods of limiting the number of included studies are time and resource intensive (eg, purposive sampling [42]). Qualitative evidence syntheses aim to understand the phenomenon of interest in a context rather than aggregate data from large representative samples of studies to achieve statistical generalizability [42]; therefore, we do not anticipate this affecting the findings of this review. Third, one reviewer performed the full screening and data charting. We minimized the potential for increased errors and lower reproducibility because of this by piloting forms, estimating interrater reliability, and consulting with the wider research team. Multimedia Appendix 7 [25,38,40-46] provides more details on our streamlined approach.

## Results

### Study Selection

**Figure 1:**
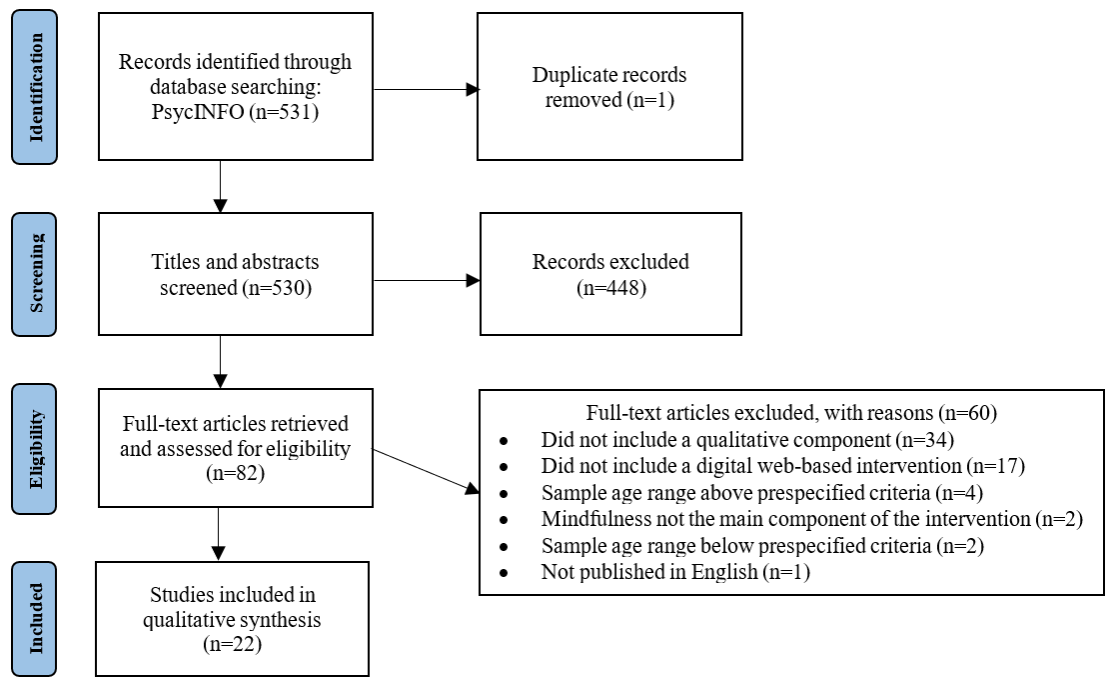
PRISMA (Preferred Reporting Items for Systematic Reviews and Meta-Analyses) flowchart for identification and selection of studies.

The searches identified 530 unique records. Of these 530 records, 82 (15.5%) were included in the full-text review and 22 (4.2%) were included in the qualitative synthesis (Figure 1). We performed the first search on September 13, 2021, and reran the search on November 30, 2021, before analysis (Multimedia Appendix 8 [35,47-51]).

### Study Characteristics

#### Overview

Detailed characteristics of the included studies are presented in Table 1. An overview of these characteristics is provided in the following sections.

**Table 1:** Overview of the included studies.

Author, year of publication (country^a^)	Sample characteristics and recruitment	Intervention description	Additional support	Data collection and analysis methods^b^
Berg and Perich [52], 2022 (Australia)	N=726; 552 (76%) female, 151 (20.8%) male, 23 (3.2%) other; aged 18-30 (mean 21.61, SD 3.45) years; young adults with different levels of depression severity (no depression, mild to moderate, and severe to extreme); 230 (31.7%) had used MMAs^c^ before. Recruited via University of Sydney participant pool, Mechanical Turk, social media, and word of mouth.	Commercially available MMAs: Headspace (43.9%), Calm (18.7%), Smiling Mind (9.1%), other (21.7%), and unspecified (6.5%)	None.	Questionnaire^d^; descriptive or inferential statistics. Participants listed their reasons for and against using MMAs in optional “other” response categories following checklist items.
Boggs et al [53], 2014 (United States)	N=38; 27 (71%) female, 11 (29%) male; mean age 46.89 (SD 12.38) years; 1 (3%) Asian, 1 (3%) African American or Black, 34 (89%) White, 2 (5%) other; individuals with a history of ≥1 major depressive episode but not currently experiencing moderate to moderately severe levels of depression. Recruited from medical settings via letters, flyers, and referrals.	Mindful Mood Balance (MBCT^e^): 8 × 60-90–minute weekly sessions; prerecorded meditation audio and videos of an in-person MBCT group, prewritten reflective questions, downloadable content, home practice, group “ask a question” function, support contact, and reminders	Support person introduced self within 48 hours, oriented participants to the intervention, welcomed participants to each session, guided participants through the content, answered questions (whole group and individually), and provided personal reminders via phone or email.	Interviews; content analysis. Participants gave feedback on website components, program content and delivery, and skills learned.
Chittaro and Vianello [54], 2016 (Italy)	N=15; 10 (67%) female, 5 (33%) male; aged 22-29 (mean 25.47, SD 2.39) years; individuals with no or minimal experience with meditation. Recruited via direct contact.	AEON mindfulness app: daily practice over 5 weeks; participants wrote thoughts and worries in the app and practiced decentering from thoughts by watching them disappear like ripples in water; support contact	Support person answered questions (individually).	Interviews; thematic analysis. Participants gave feedback on what they thought and felt while using AEON and how to improve it.
Compen et al [55], 2017 (the Netherlands)	N=31; 6 (19%) male; mean age 53.0 (SD 12.3) years; patients with cancer who experienced at least mild psychological distress; 14 (45%) completed, 10 (32%) did not complete, and 7 (23%) did not start eMBCT^f^. Recruited via web-based media, patient associations, and mental health care centers.	MBCT (for patients with cancer): 8 weekly sessions and 1 full-day silent retreat; each session contained introductory text, guided audiotaped exercises, and diaries; home practice; feedback; and support contact	Support person sent welcome message, provided personal written feedback (asynchronous), and answered questions (individually).	Interviews; content analysis. Participants gave feedback on how they experienced the eMBCT, what facilitated and impeded their participation, and how to improve the intervention.
Felder et al [56], 2017 (United States)	Women (N=37); mean age 30.49 (SD 4.09) years; 1 (3%) Asian, 2 (5%) African American, 32 (87%) White, 2 (5%) other; individuals who were currently pregnant and had a history of ≥1 major depressive episode but did not currently meet the criteria for a major depressive episode. Recruited from the community via web-based resources, flyers in medical and retail settings, and direct referral from obstetric care providers.	*Mindful Mood Balance* (MBCT for perinatal women): 8 weeks; prerecorded videos of an in-person MBCT group, audio-guided mindfulness practices, yoga DVD, reflection questions, didactic descriptions, home practice, and optional coaching	Support person provided initial orientation call and provided optional weekly coaching calls (individually or in groups).	Interviews; thematic analysis. Participants gave feedback on their satisfaction with and experience of using the program.
Forbes et al [16], 2018 (United States)	N=169; 116 (68.6%) female, 53 (31.4%) male; aged 18-58 (mean 20.33, SD 4.44) years; 109 (64.5%) White, 26 (15.4%) African American, 5 (3%) Latino, 16 (9.5%) Asian, 13 (7.7%) other; undergraduate students interested in learning meditation and with no previous meditation experience. Recruited via university participant pool.	Mindfulness meditation program: 10 × 10-minute daily sessions (up to 30 days), guided meditation audios, and reminders	Participants were sent a reminder if 3 days passed without a log-in.	Questionnaire; descriptive or inferential statistics. Participants described obstacles to practice in a single optional open-ended question following checklist items.
Kennett et al [57], 2021 (Australia)	N=14; 100% female; aged 20-59 (mean 27.60, SD 10.42) years; individuals experiencing insomnia symptoms. Recruited via university staff and student research portal and flyers in the community.	A Mindful Way to Healthy Sleep (mindfulness-based therapy for insomnia): 6 weekly modules; videos, text, reflective exercises, meditation recordings, and quiz; reminders; and support contact	Automated email reminders with encouragement. Support person answered nonclinical and technical questions (individually).	Interviews and questionnaire; thematic analysis. Participants gave feedback on satisfaction, perceived benefit, and perceived barriers to practice.
Kerr et al [58], 2019 (United States)	N=149; 126 (84.6%) female, 23 (15.4%) male; age: 11 (7.4%) aged <26 years, 50 (33.6%) aged 18-35 years, 51 (34.2%) aged 36-45, 28 (18.8%) aged 46-55 years, 9 (6.0%) aged 56-64 years; 141 (94.6%) White, 8 (5.4%) non-White; 9-1-1 telecommunicators; 71 (47.7%) completed the intervention, and 32 (21.5%) did not complete a single session. Recruited from emergency call centers via staff announcements, recruitment emails and flyers, and word of mouth.	MBSR^g^ (for 9-1-1 telecommunicators): 7 × 20-30–minute weekly lessons; videos, text, and guided audio meditation; home practice; and discussion board	Support person moderated the discussion board.	Questionnaire; thematic analysis. Participants gave feedback on how they practiced, perceived effects, what they liked and disliked about the training, and anything else about their experience.
Kubo et al [34], 2021 (United States)	Women (N=20); aged 19-39 (mean 31.4) years; 2 (10%) African American or Black, 3 (15%) Hispanic, and 13 (65%) White, 1 (5%) Asian, 1 (5%) Multiracial; pregnant women with moderate to moderately severe depressive symptoms without a regular mindfulness or meditation practice. Recruited via obstetrics and gynecology clinics.	Headspace: 10-20 min daily over 6 weeks; 30-day “Basics” course, then choice of other situation-specific courses; audio and video; and reminders	Optional push notification reminders. Support person called participants to remind them to engage if they did not complete ≥3 sessions in the previous week.	Interviews; thematic analysis. Participants gave feedback on their experience with and recommended changes to the study and Headspace.
Levin et al [59], 2017 (United States)	N=79; 52 (65.8%) female; 70 (88.6%) White, 2 (3%) Asian, 2 (3%) Hispanic, 2 (3%) multiracial, 1 (1%) African American, 1 (1%) Native Hawaiian or other Pacific Islander; mean age 20.51 (SD 2.73; mode 18) years; college students. Recruited via university participant pool, in-class talks, flyers on campus, and student health centers.	ACT^h^ website: 6 sessions over 4 weeks; text, audio, and videos; worksheets and assessments with feedback; home practice; and reminders	Support person provided tailored feedback and gave personal reminders (via email and phone).	Questionnaire; thematic analysis. Participants responded to a single item asking what they liked the least about the program.
Monshat et al [35], 2012 (Australia)	N=13; 8 (61.5%) female; aged 16-26 (mean 22) years; Australian young people. Recruited via a youth mental health promotion website.	Mindful Awareness Training and Education: 6 weeks; prerecorded videos of a live training group, guided audio meditation, discussion forum, and home practice	Support person answered questions (whole group) and contributed to the discussion forum.	Interviews; thematic analysis. Participants gave feedback on the ways in which specific aspects of the study and intervention could be improved.
Monshat et al [47], 2013 (Australia)	N=11; aged 16-24 years; healthy young people; 8 (72.7%) completed the program. Recruited via posters at a local university, welfare officers in local high schools, and a youth mental health promotion website.	Mindful Awareness Training and Education: 6 × 90-minute weekly sessions; prerecorded videos of a live training group, guided audio meditation, discussion forum, and home practice	Support person answered questions (whole group) and contributed to the discussion forum,	Interviews, focus groups, and questionnaire; grounded theory. Participants gave feedback on their understanding and experiences of mindfulness practice.
Osin and Turilina [60], 2022 (Russia)	N=175; 140 (79.9%) female; aged 18-67 (mean 30.08, SD 8.78) years; novice meditators. Recruited via social networks.	Mindfulness meditation program: approximately 12.8 min daily over 3 weeks; guided audio meditation, reminders, and support contact	Daily SMS text message reminder. Support person responded to questions, problems, and suggestions (individually) and did not provide coaching or feedback.	Questionnaire; content analysis. Participants shared their experiences during meditation or difficulties they faced in a single optional item.
Price-Blackshear et al [48], 2020 (United States)	Couples MBI^i^: Women (n=36); mean age 39.67 (SD 5.44) years; 32 (88.8%) European American or White, 2 (5.6%) African American or Black, 1 (2.7%) Asian American, 1 (2.7%) Multiracial American. Individual MBI: Women (n=41); mean age 38.78 (SD 5.08) years; 33 (80.5%) European American or White, 2 (4.9%) African American or Black, 2 (4.9%) Asian American, 1 (2.4%) Hispanic American, 2 (4.9%) Multiracial American. All women diagnosed with breast cancer. Recruited via clinical trials, referrals, and breast cancer registries and support groups.	Couples MBI vs individual MBI: 8 × 60-minute weekly prerecorded videos of trained MBSR and mindfulness-based relationship enhancement teachers, guided meditation audios of 10-30 minutes, reminders, and support contact	Participants received 2 weekly reminders. Support person answered questions about content or participation via email or phone (individually).	Questionnaire; descriptive or inferential statistics. Participants gave feedback on acceptability (eg, whether they wanted more contact with the instructor or participants).
Reyes [61], 2022 (United States)	N=23; 14 (60.9%) male, 9 (39.1%) female; aged 23-43 years (mean 31.2, SD 5.5); college student veterans with PTSD^j^ symptoms. Recruited via email to university military and veteran services.	ACT app: 4 weeks; daily audio-guided meditations, weekly videos, reflection journals, weekly phone check-in, and reminders	Push notification reminders. Support person provided weekly check-in calls.	Interviews; grounded theory. Participants gave feedback on their experience in learning and developing mindfulness.
Reyes et al [62], 2020 (United States)	N=23; 14 (60.9%) male, 9 (39.1%) female; aged 23-43 years (mean 31.2, SD 5.5); college student veterans with PTSD symptoms. Recruited via email to university military and veteran services.	ACT app: 4 weeks; daily audio-guided meditations, weekly videos, reflection journals, weekly phone check-in, and reminders	Push notification reminders. Support person provided weekly check-in calls.	Interviews; thematic analysis. Participants gave feedback on perceived benefits, facilitators of and barriers to use, and ideas for improvement.
Stjernswärd and Hansson [63], 2017 (Sweden)	N=78; 70 (89.7%) women, 8 (10.3%) men; age: 5 (6.4%) aged 20-29 years, 4 (5.1%) aged 30-39 years, 20 (25.6%) aged 40-49 years, 25 (32.1%) aged 50-59 years, 18 (23.1%) aged 60-69 years, and 6 (7.7%) aged ≥70 years; relative or significant other of a person with a mental illness and no previous experience of mindfulness meditation. Recruited via advertisements in papers, newsletters, on the web, social media, and interested clinics and organizations.	MBSR (for families living with mental illness): 2 × 10 minutes/day, 6 days/week for 8 weeks (10-week test period); audio, video, text, time log, and a private diary; reminders; and support contact	Participants received weekly email reminders. Support person provided technical support.	Questionnaire; content analysis. Participants expanded on checklist items about usability in optional free-text answers.
Stjernswärd and Hansson [64], 2017 (Sweden)	N=15; 14 (93.3%) women, 1 (6.7%) men; aged 26-69 (mean 51) years; relative or significant other of a person with a mental illness and no previous experience of mindfulness meditation. Recruited via advertisements in papers, newsletters, on the web, and interested organizations.	MBSR (for families living with mental illness): 2 × 10 minutes/day, 6 days/week for 8 weeks (10-week test period); audio, video, text, time log, and a private diary; reminders; and support contact	Participants received weekly email reminders. Support person provided technical support.	Interviews and questionnaire; content analysis. Participants gave feedback on their experiences of using the program (eg, its usability, motivators of and barriers to use, and ideas for improvement).
Stjernswärd and Hansson [65], 2020 (Sweden)	N=10; 9 (90%) women, 1 (10%) men; aged 25-73 (mean 57.6) years; relative or significant other of a person with a mental illness and no previous experience of mindfulness meditation. Recruited via advertisements in papers, newsletters, on the web, social media, and interested clinics or organizations.	MBSR (for families living with mental illness): 2 × 10 minutes/day, 6 days/week for 8 weeks (10-week test period); audio, video, text, time log, and a private diary; reminders; and support contact	Participants received weekly email reminders. Support person provided technical support.	Interviews; content analysis. Participants gave feedback on program content; format; potential effects; and motivators, hindrances, and general experience of use.
Trub and Starks [66], 2017 (United States)	N=29; 27 (93.1%) female, 1 (3.4%) male, 1 (3.4%) transgender male; mean age 25.59 (SD 3.61) years; 3 (10.3%) Asian or Asian American, 3 (10.3%) Multiracial, 23 (79.3%) White; young adults who engage in potentially risky smartphone-related behaviors. Recruited via advertisements through email, social media, and on the web news magazine.	Mindful Messaging app: daily lessons over 3 weeks; audio recordings, check-in questions, and reminders	Reminders throughout the day.	Questionnaire; descriptive or inferential statistics. Participants elaborated on ratings on the helpfulness, challenges, and effects of the program in optional open-ended textboxes.
Walker et al [67], 2010 (United States)	Focus group: n=9; 7 (77.8%) female; 2 (22.2%) Black, 7 (77.8%) White; mean age 33.56 (SD 10.69) years. Questionnaire: n=53; 40 (83%) female; mean age 35.08 (SD 10.74) years; 13 (27%) Black, 35 (73%) White. All diagnosed with epilepsy and experiencing current depressive symptoms (but not severe depression). Recruited from a hospital-based epilepsy clinic.	Project UPLIFT^k^ (MBCT for people with epilepsy): 8 × 1-hour sessions; video lessons, audio meditations, discussion board, check-ins, and home practice	Support person posted to the discussion board, answered questions (individually), and contacted participants following inactivity to check in.	Focus groups and questionnaire; thematic analysis. Participants gave feedback on materials and their experience (eg, what they liked, disliked, and would change).
Yu et al [68], 2021 (China)	N=30; 17 (56.7%) female, 43.3%) male; aged 33-68 (mean 49, SD 10.39) years; patients diagnosed with ankylosing spondylitis. Recruited from 2 local nonprofit organizations.	Pain management program (for people with ankylosing spondylitis): 5 weekly chapters, text and videos, practice time log, and reminders; each participant was followed up by a counseling psychologist	Support person offered advice and assistance, reviewed individual progress, assigned the next activity, and sent reminders.	Focus groups; thematic analysis. Participants gave feedback on effects, difficulties in practicing, and ideas for improvement.

^a^Country of institutional affiliation of the first author.

^b^Data collection and analysis methods for data included in the qualitative evidence synthesis.

^c^MMA: mobile mindfulness app.

^d^Self-completion questionnaire with open-response categories.

^e^MBCT: mindfulness-based cognitive therapy.

^f^eMBCT: internet-based mindfulness-based cognitive therapy.

^g^MBSR: mindfulness-based stress reduction.

^h^ACT: acceptance and commitment therapy.

^i^MBI: mindfulness-based intervention.

^j^PTSD: posttraumatic stress disorder.

^k^UPLIFT: Using Practice and Learning to Increase Favorable Thoughts.

#### Year and Country

The 22 studies were published between 2010 and 2022, with most (n=17, 77%) published from 2017 onward. The studies were primarily from the United States (11/22, 50%), Europe (6/22, 27%), and Australia (4/22, 18%). Multimedia Appendix 9 contains details on the years and countries.

#### Participants

The target population included students (2/22, 9%); young adults (4/22, 18%); individuals with no meditation experience (2/22, 9%); relatives or significant others of a person with mental illness (3/22, 14%); 9-1-1 telecommunicators (1/22, 5%); and individuals with symptoms, a diagnosis, or a history of a psychological disorder or another health concern (10/22, 45%). Some studies (6/22, 27%) had samples with a combination of these characteristics. Overall, the samples were approximately 78% female and 79% White, and participants were aged between 16 and 69 years. Using data from 86% (19/22) of studies that reported or from which we could calculate the mean sample age, the weighted average was 26.4 (weighted SD 8.8) years.

#### Interventions

The digital interventions tested included mindfulness-based stress reduction or mindfulness-based stress reduction tailored to families living with mental illness (5/22, 23%); MBCT or MBCT tailored to patients with cancer, the perinatal period, or people with epilepsy (4/22, 18%); acceptance and commitment therapy (3/22, 14%); commercially available mindfulness programs (2/22, 9%); and other mindfulness-based programs (8/22, 36%). Additional support to facilitate intervention completion was included in all but one study (21/22, 95%). This ranged from automated reminders and nonclinical (ie, purely technical) assistance to orientation calls and coaching. At least 86% (19/22) of the studies included human (vs automated) support, and at least 55% (12/22) of the studies included support that went beyond purely technical or administrative assistance (eg, clinical or psychologically active guidance).

#### Outcomes

In the intervention studies, the most commonly used outcome and process measures were mindfulness; psychological flexibility; and variables related to mental health, including depression, anxiety, stress, and well-being.

#### Methods

Most studies used in-depth interviews (12/22, 55%) or self-completion questionnaires with open-response categories (12/22, 55%) to collect data, whereas other studies (3/22, 14%) used focus groups. The studies primarily used thematic analysis (10/22, 45%) to analyze the data, but other methods included content analysis (6/22, 27%), descriptive or inferential statistics (4/22, 18%), and grounded theory (2/22, 9%).

### Critical Appraisal

All studies were assessed as adequately reported (Multimedia Appendix 10 [16,34,35,47,48,52-68]), including qualitative studies (8/22, 36%) and mixed methods studies or questionnaire surveys when evaluated both as a whole and with respect to qualitative data on user experience only (14/22, 64%). Overall, each study reported on the study design and questions, participant selection, data collection, and analysis. When we evaluated mixed methods studies or questionnaire surveys considering only data included in our qualitative evidence synthesis, 32% (7/22) of the studies did not provide details of the analysis method (eg, the authors reviewed open responses for common themes without reference to or full description of the method), and 5% (1/22) of the studies did not describe data collection sufficiently.

### Qualitative Synthesis

We identified three themes: (1) *responses to own practice*, (2) *making mindfulness a habit*, and (3) *leaning on others*. Each theme is outlined in the following sections using illustrative quotes.

#### Responses to Own Practice

A predominant theme was that negative reactions to one’s own application of mindfulness during digital MBIs are common and can discourage continued efforts. Participants reported not being able to practice at times, either because they could not find time to practice or because they experienced distractions that interrupted their practice. When participants experienced difficulties in scheduling time to practice, they also expressed feelings of guilt, resentment, and self-criticism, which depleted their motivation and led them to view practice as another stressful demand:

I am finding it is almost causing more stress trying to find the time to get practice in and to do the weekly lessons.

Participant [58]

[I felt] a little critical of self, felt like I couldn’t do it all, and it was my fault somehow, and this is too much to ask with your daily life, and resentful.

Participant [53]

Similarly, participants felt frustrated by disturbances originating from their environment (eg, shared spaces and noise levels) and internal experience (eg, negative emotions, life problems, and daily plans):

With project deadlines in parallel it is hard to choose a time for meditation, very angry at myself.

Participant [60]

At times, there were too many interruptions that I would get frustrated.

Participant [58]

In addition to not being able to practice at times, participants’ preoccupation with “doing it right” also fueled negative reactions to their practice, which reduced motivation and expectations of benefit. There was a repeated idea that there is a right way to practice, and this was often expressed in the form of insecurity about practicing properly. Participants reported not knowing what was expected of them or what should happen during practice, feeling puzzled and confused by the effects they experienced and questioning the accuracy of their training (eg, when they fell asleep, whether brief practices “count” or they had “permission” to do a briefer practice when short of time, or whether they were in the correct position):

I always want to do things right, and I wasn’t sure about how I did the meditation exercises in the beginning. Is this the way I am supposed to do this?

Participant [55]

When I listen, I have a feeling that I do not quite understand what should happen during the meditation.

Participant [60]

Not knowing exactly what was expected in terms of program structure and training dose (despite information), and lack of adherence towards the recommended dose sometimes induced a sense of insecurity as to whether one was doing the training properly and actually benefiting from it or taking it seriously enough. This could deplete motivation. [Author]

Participant [65]

I’m worried whether I am doing the practices correctly.

Participant [56]

It’s really good to have that permission, so to say. I did do the 3-minute breathing space a few times, but I guess I was thinking that wasn’t really doing the homework because it is so brief. It’s good to know that “counts.”

Participant [56]

This led to the desire for feedback on whether participants had performed training properly and an additional brief “overview” tutorial to aid memory in instances of insecurity [65].

#### Making Mindfulness a Habit

Another prominent notion was that establishing a consistent training routine is not only an essential part of digital MBIs but also one that requires resolution, perseverance, and self-discipline. Participants recognized that being successful in creating a routine and integrating mindfulness into their lives made regular practice easier and that regular practice was important when learning a new skill such as mindfulness:

It was difficult in that you had to carve out the time really consistently, but it was also really valuable. I don’t think the program would be as effective if you weren’t being asked to do it daily. What I understand is you’re trying to develop a habit.

Participant [53]

To manage the issue of dwindling enthusiasm, the participants made two suggestions. First, it was important to practise more to make it become a natural habit. [...] setting aside time each day for lying down and practising the exercises before sleep and even during the daytime whenever possible, no matter how short the exercise was, could help them build up their perseverance.

Author [68]

However, seeing the value of making mindfulness a habit was not enough to meet the responsibility. Participants reported needing to persist and grapple with the effortful task of making practice a scheduled activity, which involved frequent adjustments to their plans, priorities, and commitments:

You just have to make time for it like you make time for anything else you want to do. You just have to work for it if this is something that you want.

Participant [62]

It’s a question of discipline /.../ I think one should pinpoint that it’s strenuous and that one has to be ready to struggle with it because one believes in it.

Participant [64]

As the participants began to accommodate the daily use of the app into their already busy personal, academic, and professional schedules, they encountered the challenges of establishing a new habit. For the participants, this was not a straightforward process, but rather involved several adjustments in their schedules, priorities, obligations.

Author [62]

In addition to having self-discipline and an inner resolution, identifying a designated space to practice or connecting practice with an existing routine activity, such as brushing teeth or taking medication, helped participants get into the habit:

I made it important to always do it like in the same place in my apartment and like around the same time. I just have a chair in my living room, and I always did it in that chair. So yeah, it was always the same chair. The same with the lighting, it would be the same lights which were turned on. Like every day, the situation was pretty much always the same. However, there are lots of distractions in my life, so that’s why I am still basically kind of baking it [meditation routine] into like a scheduled activity.

Participant [61]

To make home practice engagement more likely three interviewees suggested asking participants to practice at the same time every day perhaps “pegging it” to a routine activity (e.g., after brushing their teeth in the morning) [...] Another suggested drawing a parallel with the ritual and regularity of “when you’re on a medication” when describing the approach to practice.

Author [35]

Participants also highlighted the need for personalization (ie, the provision of content that is tailored to the needs and preferences of individual users) to motivate individuals to embed mindfulness into their lives. For example, some participants preferred shorter practices as they were more attainable with respect to remaining attentive (ie, minimizing interruptions and loss of focus), scheduling (ie, easier to make time for and integrate into daily life), and avoiding adverse experiences (ie, boredom, impatience, and discomfort from sitting still), whereas others preferred longer sessions that allowed time for the mind to slow down and for participants to concentrate better. Such contradictory preferences extended to several aspects of the intervention (eg, the amount of narration during guided meditations, format of content delivery, degree of variation in subject matter, and frequency of reminders), and participants appreciated when they were considered:

I liked that there was a variety of practices to try. Different things work for different people and that was taken into account.

Participant [58]

Qualitative data revealed vast individual differences in the preferences for meditation. Voice instructions appeared helpful to some and disturbing to others; the same meditation sessions were experienced as being too short or too long; some participants enjoyed the soft background sounds of nature while others said they would have preferred some background music; some individuals were frustrated by the silent pauses that others appreciated and enjoyed; some were uncomfortable with the same themes and practices found to be particularly helpful by other participants. All of this [...] suggests that “one-size-fits-all” online interventions might be less engaging and less effective than those tailored to individual preferences.

Author [60]

#### Leaning on Others

A core idea expressed in various ways throughout the data set was that those engaging with digital MBIs depend on someone else—whether a therapist, researcher, significant other, or another participant—for support and encouragement and that this improved engagement. An aspect of this idea was that receiving any form of communication from the digital MBI (eg, automated reminders; messages of encouragement; or personalized feedback via email, SMS text message, or phone call) was helpful in reminding and motivating participants to practice without feeling intrusive:

A consistent message from all interviewees was that any form of feedback or communication from the programme was likely to improve retention. In addition to forms of feedback already mentioned, email (even if automated and using a “no-reply” address), and text message reminders, were thought to be likely to be helpful without being intrusive.

Author [35]

I enjoyed the reminders that the app sends you—I really found that helpful because otherwise, I would not have remembered to do it.

Participant [62]

Similarly, having a program “support person” was considered essential. Many valued the existence of an individual (eg, instructor, coach, therapist, or member of the research team) with whom they could discuss program concepts and from whom they could receive technical or administrative support. Participants felt that it was reassuring to know someone was available if needed, whether via phone, email, or an “Ask a Question” or “Help” function:

All participants saw the value of having a support person available who was only a phone call or email away. Some participants mentioned more frequent interactions with the support person and even those who did not use the support reported that it was an important asset of the program.

Author [53]

Many endorsed that it was “essential” to have a coach and helpful to know that one was available if needed.

Author [56]

Another main expression of this theme was not feeling part of a community, which led participants to feel alone or that they lacked a connection or sense of belonging with other users. This in turn motivated requests for a “community component” (eg, web-based forums, message boards, or group [video or phone] chats) so that participants could discuss their intervention experiences, clarify content, and share challenges with other users. This was particularly desired by participants with a shared lived experience so that they could interact, connect, and identify with others (eg, perinatal women, individuals with epilepsy, or patients with cancer). Although most of the included studies (16/22, 73%) were of interventions that did not have a community component, this component was also highly valued by participants for whom it was present (6/22, 27%):

I think this would be a lot better if there was a Web-based group...I felt alone out here. I would have been engaged more.

Participant [53]

All interviewees agreed that an online forum, which enabled discussion about their programme experiences, was highly desirable and was likely to boost retention significantly through: clarifying aspects of the teaching; sharing and overcoming difficulties with practice; and encouraging participants to remain engaged and complete home practice sessions.

Author [35]

The majority expressed [...] a desire for a community function component of the program that would allow them to interact with other perinatal women who were using MMB [Mindful Mood Balance program].

Author [56]

A final dimension captured the tendency of participants to engage in creative ways to seek support from others when none or not enough was provided by the program. Participants reported sharing the program with significant others, such as family members, friends, and spouses, to help encourage their consistent and continued practice:

I’m talking to my husband about how he can help me protect some time on the weekends to do the longer practices.

Participant [56]

My kids actually started to look forward to it, so they would actually ask to do it. That helped me kind of stay on track.

Participant [62]

Some participants were open with their training, sharing their experiences with the patient and family members and occasionally doing some of the exercises together.

Author [65]

By reaching out to others in their lives, participants were able to orchestrate their own social environment to support their engagement with the program. This self-made way of forging a helpful foundation for practice not only highlights the impact that someone else can have on people’s engagement with digital MBIs but also indicates that people are not reliant on a mindfulness teacher to feel supported.

## Discussion

### Principal Findings

This review identified, critically appraised, and synthesized qualitative data from 22 original studies of people’s experiences using a digital MBI to identify factors that facilitate or act as barriers to their engagement with the intervention. Three overarching themes appeared to influence engagement: (1) *responses to own practice*, (2) *making mindfulness a habit*, and (3) *leaning on others*. Together, these themes provide crucial insights as to why people frequently stop engaging with digital MBIs. The following discussion elaborates on these areas and offers some recommendations for researchers and developers to guide intervention design and evaluation, thereby improving acceptability and engagement with digital MBIs, increasing their effectiveness, and supporting their translation to real-world use.

The first theme emphasized how adverse reactions to one’s own practice are common and may serve to reduce motivation. This suggests that the tendency to respond negatively to one’s own experience and application of mindfulness is a major barrier to using digital MBIs, which is consistent with the wider literature on mindfulness interventions and offers initial support for extending this finding to digital intervention formats. For example, in one study, the question “Am I doing it right?” emerged by the second week of a traditional MBCT course [69]. In another study, participants reported feeling self-critical when they could not make time to practice and when mindfulness did not appear to work for them [18]. As in this review, this negative reaction made it difficult for participants to continue to engage, prompting them to give up and remove it from their to-do list. To help overcome this barrier, traditional face-to-face programs such as MBCT explicitly allocate time to anticipating what difficulties and obstacles may arise in doing home practice (eg, trying to find free time) and how to deal with them [7]. Such content on overcoming barriers may be lost in the translation to digital formats, and our review is the first to highlight the importance of explicitly addressing this in digital MBIs.

This finding also indicates that one of the most important factors influencing engagement with digital MBIs is unique to mindfulness specifically rather than general to digital interventions and reflects the metacognitive nature of the intervention’s target behavior. Our review offers clear guidance on which particular combinations of factors identified across other literature (eg, on digital interventions or mindfulness interventions more broadly) are most influential in the specific context of digital MBIs, which is essential to make these interventions more persuasive, feasible, and relevant to users [20].

The second theme (*making mindfulness a habit*) highlighted the need and effort required to practice consistently and a call for personalization to help achieve this. This suggests that forming a mindfulness habit is a key barrier to sustained engagement with digital MBIs and that persuasive technological features could help overcome this barrier. Although prior work on digital interventions has identified personalization as an important feature, this review is the first to demonstrate its relevance to digital mindfulness interventions specifically. For example, a systematic review of web-based interventions found that the inclusion of persuasive design principles, including tailoring (ie, provision of content or feedback adapted to factors relevant to a user), explained 55% of the variance in session completion across studies [70]. Our findings suggest that certain factors that contribute to engagement with digital content in mobile and web-based interventions more generally may also apply to interventions for which engagement with the digital content is designed to facilitate completion of a nondigital target behavior (eg, an experiential mindfulness exercise) [11]. Notably, the threshold of engagement with the digital component that successfully facilitates the “non-digital target behaviour” can demonstrably vary between individuals [71], supporting a shift toward patient-treatment matching and person-centered care [72] and underscoring the need to implement this digitally (eg, through automated personalization).

Conversely, this theme diverges from the results of a thematic analysis of the experiences of health care professionals who participated in either a web-based or printed self-help MBI [18]. The health care professionals consistently reported that longer practices were more challenging to engage with than shorter practices, whereas our review found considerable variation in preferences for different intervention features (eg, format, materials, and sound), including length of practice, perhaps because of the breadth of MBIs included in our robust evidence synthesis. This highlights the importance of understanding the key behavioral and psychological needs of the target population to ensure that the intervention addresses them.

The third theme (*leaning on others*) highlighted that those engaging with digital MBIs are encouraged by additional support in its broadest sense (ie, *any* communication designed to support *any* aspect of the intervention, its completion, or its desired outcomes). This includes synchronous (eg, phone calls and web-based chats) and asynchronous (eg, email and SMS text messages) communication, support provided to a group of people (eg, discussion forums and group chats), and anything else (eg, automated reminders, technical assistance, feedback, and reaching out to someone). Although these results align with those of previous research on the impact of additional support in digital interventions [73], this study cannot draw conclusions on the relative power of each type of support because of the variability across studies. Given this, the provision of support in research settings needs to be considered. Interventions from almost all the studies in this review included additional support; however, it was not always clear what this constituted. For example, some studies (2/22, 9%) reported that participants could ask questions via email but did not specify whether they received clinical or purely technical assistance. Relatedly, participants may not have used the support on offer, although the results from this review indicate that this is not as important as having it available. Additional support in other studies (3/22, 14%) was provided to a group of participants; however, this type of support has been excluded from definitions of guidance [74]. Future research could explore whether there are unique barriers to engagement in guided versus unguided digital MBIs and compare different types and levels of support to advance understanding of how, when, and for whom additional support can improve engagement. This is important as there is a trade-off between the provision of support and scalability—if digital MBIs need to have someone always available to be engaging, they will be limited in reach and cost-effectiveness.

Irrespective of these uncertainties regarding the relative contributions of different types of support, it is worth noting that social support was found to be a key facilitator of engagement. This idea is consistent with the historical origins of mindfulness (ie, to be practiced collectively and in community [75]) and findings from in-person group settings. In a synthesis of the accounts of individuals with mental health difficulties in group MBIs [76], learning mindfulness within a group was found to be helpful as peer support encouraged perseverance with course demands and learning alongside people with similar experiences fostered a comfortable and destigmatizing environment. Our findings point to the idea that digital MBIs may suffer decreased engagement as a result of reduced social support.

### Implications for Intervention

Researchers can use the factors identified in this review to guide intervention design and, ultimately, improve engagement with digital MBIs. However, such strategies must be (1) grounded in relevant literature and (2) directly relevant to the individuals who will use the interventions. For example, the second theme suggests that instructing people to practice regularly is unlikely to turn it into a habit. Researchers might consider drawing on research on behavior change and habit formation, particularly with regard to digital interventions (eg, gamification technology to motivate behavior change). Researchers might also consider carrying out primary qualitative research to ensure that the generated strategies are informed by and meet the priorities and needs of the intended user. The person-based approach offers a systematic means of integrating theory, evidence, and user perspectives into initial intervention planning [19,20]. Therefore, the themes highlighted in this review could inform the production of guiding principles within this approach (ie, intervention design objectives and key features intended to achieve each aim).

### Strengths and Limitations

To the best of our knowledge, this is the first review to synthesize qualitative evidence from individual studies across different contexts to advance the understanding of the barriers to and facilitators of engagement with digital MBIs. Using inductive thematic synthesis encouraged the generation of themes that “go beyond” the content of the primary studies to produce novel findings. All 22 studies were assessed as being adequately reported, which suggests that the papers included in this review are of sufficient quality to draw concrete inferences. We also followed established methodological guidance; used an a priori published protocol; and took several steps to increase the validity and reliability of the review, including pilot-testing forms and procedures, consultation with an information specialist, and regular team meetings.

In terms of limitations, we restricted our search to PsycINFO to manage the number of studies in a resource-efficient manner. However, it is possible that this led to the omission of additional relevant studies or introduced selection bias. Where possible (eg, in reviews with longer time frames), researchers should consider searching several sources and using purposive sampling to ensure that the final set of included studies meets relevant criteria (eg, has a wide geographic spread or rich data [42]). The studies included in this review reported mostly on White adult female individuals from Western countries, which means that the generalizability of our findings to underrepresented groups is unclear. This is an important area for further research as initial engagement with digital and mobile health interventions is lower in some underserved populations (eg, people of lower socioeconomic status [28]). Relatedly, we excluded studies with samples entirely aged <16 years and/or >35 years because of the focus of our own intervention development being on young people. Although the final age range covered was 16 to 69 years, future research would benefit from investigating engagement in younger and older populations as motivations to use digital interventions may vary.

There was significant heterogeneity across the interventions (eg, commercially available programs, acceptance and commitment therapy, mindful messaging, and guided mindfulness meditations) in the included studies, and these differences may have influenced engagement. Researchers and developers of digital MBIs should also consider how specific elements (eg, content, mode of delivery, and provision of support) might make people more or less likely to stop using the technology. Finally, although this review synthesized evidence from diverse study types, it is worth bearing in mind that engagement with MBIs is usually defined in terms of intervention use (ie, *physical* engagement [77]). It is unclear whether the factors identified in this review characterize facilitation and hindrance of aspects of *psychological* engagement, such as *intention* to practice mindfulness, *belief* that practicing mindfulness will be helpful, and *commitment* to integrating mindfulness into daily life. This is an important area for further research given evidence that psychological rather than physical disengagement from self-help MBIs has a greater impact on cultivating mindfulness [77].

### Conclusions

Previous studies have shown the potential of digital MBIs to improve a range of health outcomes. Sufficient engagement with these interventions is required to achieve the intended effects; however, engagement is typically poor. This review synthesized evidence from studies on digital MBIs and identified 3 key factors that influence user engagement. We recommend that researchers generate their own solutions to these challenges by drawing on relevant literature and working with people from the target user population.

## Appendix

Multimedia Appendix 1 PRISMA-ScR (Preferred Reporting Items for Systematic Reviews and Meta-Analyses extension for Scoping Reviews) checklist.

Multimedia Appendix 2 Piloting screening.

Multimedia Appendix 3 Data charting form.

Multimedia Appendix 4 Piloting data charting.

Multimedia Appendix 5 Excerpt from data charting.

Multimedia Appendix 6 Data analysis.

Multimedia Appendix 7 Methodological streamlining.

Multimedia Appendix 8 Study selection.

Multimedia Appendix 9 Study characteristics.

Multimedia Appendix 10 Critical appraisal.

## Abbreviations

MBCT: mindfulness-based cognitive therapy
MBI: mindfulness-based intervention
PRISMA-ScR: Preferred Reporting Items for Systematic Reviews and Meta-Analyses Extension for Scoping Reviews

## Data Availability

Relevant data are available in this paper and its supplementary files. Requests for additional material should be addressed to the corresponding author.
