# Correction: Experiences of Using Digital Mindfulness-Based Interventions: Rapid Scoping Review and Thematic Synthesis

**DOI:** 10.2196/60967

**Published:** 2024-05-29

**Authors:** Emma Louise Osborne, Ben Ainsworth, Nic Hooper, Melissa Jayne Atkinson

**Affiliations:** 1 Department of Psychology University of Bath Bath United Kingdom; 2 School of Psychology University of Southampton Southampton United Kingdom; 3 School of Psychology Cardiff University Cardiff United Kingdom

In “Experiences of Using Digital Mindfulness-Based Interventions: Rapid Scoping Review and Thematic Synthesis” (J Med Internet Res 2023;25:e44220) the authors noted one error.

In the “Results” section of the Abstract, the following sentence:

The search identified 510 studies, 22 (4.3%) of which met the inclusion criteria.

Has been corrected to:

*The search identified 530 studies, 22 (4.2%) of which met the inclusion criteria.*

The correction will appear in the online version of the paper on the JMIR Publications website on May 29, 2024, together with the publication of this correction notice. Because this was made after submission to PubMed, PubMed Central, and other full-text repositories, the corrected article has also been resubmitted to those repositories.

