# Supplementary material for: Experiences of Using Digital Mindfulness-Based Interventions: Rapid Scoping Review and Thematic Synthesis
Source: J Med Internet Res. 2023 Sep 28;25:e44220. doi: 10.2196/44220 (PMC10570895; doi:10.2196/44220)
Supplement: Multimedia Appendix 2 [file jmir_v25i1e44220_app2.pdf]

Article title: Experiences of Using Digital Mindfulness-Based Interventions: Rapid Scoping Review and Thematic Synthesis

Journal name: Journal of Medical Internet Research (JMIR)

Author names: Emma L. Osborne, Ben Ainsworth, Nic Hooper, Melissa J. Atkinson

Corresponding author: Emma L. Osborne, Department of Psychology, University of Bath, Claverton Down, Bath, BA2 7AY, UK; Email: elo25@bath.ac.uk

Multimedia Appendix 2: Piloting Screening

Brief Overview

We conducted a pilot exercise to calibrate and test the eligibility criteria. Two reviewers independently screened a random selection of 50 titles and abstracts (10% of records) then met to discuss discrepancies and modify the criteria. The first author screened the remaining titles and abstracts to determine whether they met full inclusion criteria.

Detailed Description

We followed 10 steps during the pilot exercise: (1) training: ensure both reviewers understand the eligibility criteria; (2) discussion: thoroughly discuss the screening tool (e.g., is it unambiguous?); (3) identify records: select a random subset of the same 50 titles and abstracts (10% of records) for both reviewers; (4) screen: both reviewers screen studies in training set; (5) discussion: discuss each discrepancy and difficult decision between reviewers; (6) consensus: resolve disagreements and come to a consensus on each decision (consult a third reviewer where necessary); (7) revise criteria: clarify and add detail to the eligibility criteria considering the valuable information from disagreements; (8) agreement: formally assess initial agreement between reviewers (inter-rater reliability) using a Kappa statistic (measure of chance-corrected agreement); (9) second round: determine whether a

second round of piloting is necessary; and (10) begin title and abstract screening: single reviewer screens remaining titles and abstracts using the piloted and modified criteria.

We considered the following questions:

- Are the eligibility criteria expressed clearly enough?
- Are different screeners interpreting the criteria consistently (i.e., can the criteria be reliably interpreted and applied)?
- Do the criteria distinguish between relevant and irrelevant records? (i.e., are they too stringent, resulting in the exclusion of records that are relevant to the research question, or are they too liberal, yielding irrelevant records eligible)?

During step 7 (“revise criteria”), we made several changes to specify: (1) whether pilot studies are eligible (i.e., “may report on a full-scale or pilot-scale project”), (2) whether published peer-reviewed research protocols are eligible (i.e., must be an “empirical research article”, defined as an article reporting methods and findings of an original research study conducted by the authors of the article [32]), (3) that we are taking a non-restrictive approach to study design (i.e., “including free text from questionnaire surveys”), (4) that the title and/or abstract must include a term related to the evaluation of participants’ experience (e.g., feasibility, acceptability, usability, etc.), (5) that the intervention must be digital/online (if this is not clear from the title and abstract, include article and determine eligibility from reading the full text), (6) that mindfulness must be a primary part of the intervention, and (7) that the age range of the sample must include people between 16–35 years (if this is not clear from the title and abstract, include article and determine eligibility from reading the full text).
