# Supplementary material for: Experiences of Using Digital Mindfulness-Based Interventions: Rapid Scoping Review and Thematic Synthesis
Source: J Med Internet Res. 2023 Sep 28;25:e44220. doi: 10.2196/44220 (PMC10570895; doi:10.2196/44220)
Supplement: Multimedia Appendix 3 [file jmir_v25i1e44220_app3.pdf]

Article title: Experiences of Using Digital Mindfulness-Based Interventions: Rapid Scoping Review and Thematic Synthesis

Journal name: Journal of Medical Internet Research (JMIR)

Author names: Emma L. Osborne, Ben Ainsworth, Nic Hooper, Melissa J. Atkinson

Corresponding author: Emma L. Osborne, Department of Psychology, University of Bath, Claverton Down, Bath, BA2 7AY, UK; Email:

elo25@bath.ac.uk

Multimedia Appendix 3: Data Charting Form

**Study**	**Country**	**Study design**	**Aims/purpose**	**Sample**	**Psychosocial outcomes**
• Authors<br>• Publication year<br>• Title	• Conducted<br>• Published	• Qualitative, mixed methods, or intervention study (type)<br>• Pilot or full-scale<br>• Brief content of question(s) to evaluate user experience	• Direct (verbatim) quotations	• Sample size<br>• Recruitment details<br>• Notable characteristics	• Construct(s) measured or targeted by the intervention
•	•	•	•	•	•

**Intervention description**	**User experience**	**Other findings**
• Type, duration, comparator	• Any data related to user experience, including methodological considerations (e.g., recruitment strategies, evaluation method, modifications to the intervention)<br>• Direct (verbatim) quotations	• Useful not captured
•		•
