# Supplementary material for: Experiences of Using Digital Mindfulness-Based Interventions: Rapid Scoping Review and Thematic Synthesis
Source: J Med Internet Res. 2023 Sep 28;25:e44220. doi: 10.2196/44220 (PMC10570895; doi:10.2196/44220)
Supplement: Multimedia Appendix 4 [file jmir_v25i1e44220_app4.pdf]

Article title: Experiences of Using Digital Mindfulness-Based Interventions: Rapid Scoping Review and Thematic Synthesis

Journal name: Journal of Medical Internet Research (JMIR)

Author names: Emma L. Osborne, Ben Ainsworth, Nic Hooper, Melissa J. Atkinson

Corresponding author: Emma L. Osborne, Department of Psychology, University of Bath, Claverton Down, Bath, BA2 7AY, UK; Email: elo25@bath.ac.uk

Multimedia Appendix 4: Piloting Data Charting

Brief Overview

We performed a second pilot exercise during data charting to assess the completeness and utility of the charted data. Two reviewers independently extracted data from a full text article using a template form adapted from the example evidence table for qualitative studies developed by the National Institute for Health and Care Excellence [33]. The reviewers then considered inconsistencies and improvements to the form. For example, through piloting we decided to extract all data related to user experience (from the abstract, results, and discussion sections) as direct quotation in a single column rather than organise data into barriers and facilitators, to stay true to the raw data and avoid an additional layer of interpretation during data extraction.

Detailed Description

Two reviewers piloted data charting on a single full text article to assess the completeness and utility of the extracted data. We then met to discuss inconsistencies and improvements to the form. Our main concern related to the separate “Evidence of barriers” and “Evidence of facilitators” columns in the initial form. We recognised that organising qualitative data on user experience into barriers and facilitators would introduce an additional

layer of interpretation (e.g., our own analysis in addition to the authors' interpretations of the data in the original article). We therefore decided to extract all data related to user experience (from the abstract, results, and discussion sections) as direct (verbatim) quotation into a single column ("User experience") to stay true to the raw data.

In addition to creating a single column for user experience, we clarified and added the following details to the data extraction form: (1) in the "Psychosocial outcomes" column, extract the construct(s) measured if the article is an intervention study and the construct(s) targeted by the intervention if the article is a qualitative or mixed methods (non-intervention) study; (2) in the "Intervention description" column, provide a summary of the intervention; (3) in the "Study design" column, include the method of data collection (e.g., semi-structured interviews, participants were asked the following questions: "…") and analysis (e.g., thematic analysis); (4) use direct (verbatim) quotations in the "Aims/purpose" column for efficiency (this information will only be used to provide context for each study prior to analysis); (5) use direct (verbatim) quotations in the "User experience" column to stay true to the original data and avoid adding a layer of interpretation (i.e., through summarising) during data extraction; (6) in the "Country" column, include institutional affiliation of the first author as country "published" and where the research was based (i.e., where participants were recruited) as country "conducted"; (7) include general implications and notes for consideration in the final "Other findings reported" column; (8) merge sample size, recruitment details, and notable sample characteristics into a single "Sample" column due to the overlap in this information.
