# Supplementary material for: Experiences of Using Digital Mindfulness-Based Interventions: Rapid Scoping Review and Thematic Synthesis
Source: J Med Internet Res. 2023 Sep 28;25:e44220. doi: 10.2196/44220 (PMC10570895; doi:10.2196/44220)
Supplement: Multimedia Appendix 5 [file jmir_v25i1e44220_app5.pdf]

Article title: Experiences of Using Digital Mindfulness-Based Interventions: Rapid Scoping Review and Thematic Synthesis

Journal name: Journal of Medical Internet Research (JMIR)

Author names: Emma L. Osborne, Ben Ainsworth, Nic Hooper, Melissa J. Atkinson

Corresponding author: Emma L. Osborne, Department of Psychology, University of Bath, Claverton Down, Bath, BA2 7AY, UK; Email: elo25@bath.ac.uk

Multimedia Appendix 5: Data Charting Table Excerpt

Study<br>• Authors<br>• Publication year<br>• Title	Country<br>• Conducted<br>• Published	Study design<br>• Qualitative, mixed methods, or intervention study (type)<br>• Pilot or full-scale<br>• Brief content of question(s) to evaluate user experience	Aims/purpose<br>• Direct (verbatim) quotations	Sample<br>• Sample size<br>• Recruitment details<br>• Notable characteristics	Psychosocial outcomes<br>• Construct(s) measured or targeted by the intervention
• Monshat, K., Vella-Brodrick, D., Burns, J., & Herrman, H.<br>• 2012<br>• Mental health promotion in the Internet age: a	• Australia<br>• Australia	• Qualitative, full-scale, semi-structured interviews, thematic analysis, one-on-one.<br>• "Interviews included five open questions phrased as 'In what way can the program's [subject of interest] be improved?' Key subjects were: structure, delivery mode, homework, advertising plan and enhancement of the rate	• We aimed in this study to obtain views from young people about all three aspects (viz. use- fulness, ease of use and enjoyment) of the pro- posed MATE programme as well as the	• N=13 (data saturation judged after 9)<br>• Young people recruited via Reachout.com ("a youth-specific mental health promotion space")	• Not an intervention study, but will inform future RCT, in which the "Key outcome domains: depressive or anxious symptoms, subjective well-being and sense of functioning and

consultation with Australian young people to inform the design of an online mindfulness training programme		of young people accessing the site and participant engagement and retention in the programme."	planned evaluation process. Based on an analysis of this input, we aimed to devise a training programme likely to be acceptable to young people and effective in improving their mental health and well-being.	• Aged 16–26 years (M = 22)<br>• 60% female, 50% studying fulltime, 50% had previous mindfulness training experience, none had extensive background in meditation. 50% previous diagnosis mental disorder, all stable now, none had current substance use disorder.	mental skills (emotion regulation ability and mindfulness)".

**Intervention description**	**User experience**	**Other findings reported**
• Type, duration, comparator	• Any data related to user experience, including methodological considerations (e.g., recruitment strategies, evaluation method, modifications to the intervention)<br>• Direct (verbatim) quotations	• Useful not captured
• Draft version of the Mindful Awareness Training and Evaluation (MATE) programme based on review of literature and established face-to-face programme for students.<br>• 6-week program with following elements:<br>1. Weekly core content presented as videos	From the results section:<br>**Desirability of an online programme and ideal appearance, structure and content**<br>All interviewees commented that an online mindfulness programme would be of interest to their peers. They suggested that many young people would prefer it to a live programme as	

2. Independent ‘timeout’ practice with aid of audio sessions<br>3. Online discussion forum providing sense of community and support<br>• Initial plans for evaluation were RCT with waitlist and qualitative evaluation<br>• Initial plans for advertising were via link on reachout.com	(i) the latter would take too much time; (ii) it would involve travelling to where training was held which would be a ‘hassle’; (iii) ‘the net’s where we spend all our time anyway’ and (iv) ‘if you’re all anxious you wouldn’t want to sit with a group of people’. They saw Reachout.com as a ‘brand young people trust’ which would lend authority to a programme presented thereon.<br><br>**‘Look and feel’ of the website and structure of training modules**<br><br>Male interviewees were concerned that the demonstration site, coloured a pale green and decorated with flowers, would not be appealing to young men. They suggested using darker, bolder colours (e.g. black, yellow and dark blue). All interviewees suggested not having pictures of meditators on the site as it may communicate a religious overtone. Instead they suggested pictures of young people ‘being happy, being active and having fun’ perhaps enhanced with online animation. Two interviewees encouraged the use of slang and mobile telephone text message language (e.g. abbreviating ‘you’ to ‘u’) especially in headings.<br><br>All interviewees were in favour of information delivery in video format rather than through text or audio. Although suggesting ‘as little text as possible’ (2–3 sentences in each page) on webpages they asked that teaching material be available as a downloadable PDF file as well as videos. This could allow participants to quickly review the teaching content for the week if they wished.<br><br>All interviewees preferred a series of short videos each week rather than one long presentation. Reasons given were: young people have ‘a short attention span’ and are unlikely to remain	

	attentive to a video for more than a few minutes; young people tend to do several things at once when online (e.g. chat rooms, Facebook, instant messaging etc.) and as such are unlikely to continue viewing a video if it interferes with this process and; it allows them to progress at their own pace (e.g. take breaks between videos if needed) to go back and watch each section separately if they so desired.<br><br>To avoid boredom they suggested no more than four videos each week with a maximum duration of 10 min each (three suggested a maximum of 5 min). Two interviewees commented that participants viewing latter weeks of the programme may 'cope with longer videos' as (i) they were obviously committed and (ii) their ability to attend to material may have improved through the mindfulness practices they have been engaged with.<br><br>One interviewee suggested that the whole week's page not be longer than one mouse scroll long. Another suggested videos are placed in a horizontal line (e.g. two at a time) rather than vertically. Four interviewees suggested using 'progress bars' at the top of each page, whether a teaching module or outcome measurement, so as to orient participants, create a sense of fulfilment and encourage completion.<br><br>**'Timeout' practice**<br><br>All interviewees advised against the use of the word 'homework' as it may remind participants of their university or school homework and thus be off putting. Suggested alternatives were 'home practice', 'meditation practice', 'rest' or 'timeout'.<br><br>Interviewees suggested a maximum of 5 min of daily meditation practice not more often than twice a day. They were	

	comfortable with meditation instructions being provided in downloadable MP3 format.<br><br>To make home practice engagement more likely three interviewees suggested asking participants to practice at the same time every day perhaps ‘pegging it’ to a routine activity (e.g. after brushing their teeth in the morning). Two interviewees suggested asking participants to log their home practice and record any difficulties experienced with it in a diary. Another suggested drawing a parallel with the ritual and regularity of ‘when you’re on a medication’ when describing the approach to practice.<br><br>**Retaining participants**<br><br>A consistent message from all interviewees was that any form of feedback or communication from the programme was likely to improve retention. In addition to forms of feedback already mentioned, email (even if automated and using a ‘no-reply’ address), and text message reminders, were thought to be likely to be helpful without being intrusive.<br><br>All interviewees agreed that an online forum, which enabled discussion about their programme experiences, was highly desirable and was likely to boost retention significantly through: clarifying aspects of the teaching; sharing and overcoming difficulties with practice; and encouraging participants to remain engaged and complete home practice sessions.<br><br>Sunday morning was suggested by two interviewees as a suitable time for each weekly module of the programme to become available as ‘young people check their email and Facebook on a Sunday afternoon.’	

	Another interviewee suggested phrasing the initial registration step as an ‘application process’. She suggested that this would communicate the serious nature of their undertaking and encourage young people to persevere.<br><br>Two forms of reward were suggested by interviewees to ensure programme and outcome measure completion. First by highlighting the opportunity to benefit other young people a sense of ‘doing good’ and ‘being part of a bigger thing’ may be created. Interviewees suggested that it be emphasized early on that this was a world first programme of its kind and that a future roll out of the programme would be based on the experience of participants in this pilot version.<br><br>Second physical rewards were suggested in three forms: (i) a certificate for those who complete the programme and outcome measures which may be a desirable inclusion in résumés of participants who may be engaged in mental health or community studies or careers (ii) a token reward that all programme completers and all who finish all three rounds of outcome measures would receive (e.g. a Reachout.com arm band or sticker) and (iii) a prize draw for items of monetary value.<br><br>Interviewees commented that a higher chance to win small prizes was likely to be more motivating than a small chance to win large prizes. Suggested prizes were: movie vouchers; vouchers for electronics and music stores (esp. ones which had an online purchase option); music and application download credits; mobile telephone call credits; event ticket credits and driving lessons.<br><br>**Evaluation method**	

	All interviewees concurred that proposed demographic data and assessment domains (positive mental health, symptoms of ill health and mental skills) were appropriate and likely to seem relevant to participants. One interviewee suggested additional functional items to capture such aspects as ‘eating pattern, having a routine they are happy with, and being able to catch public transport’.<br><br>All preferred online to mailed questionnaires. Completing outcome measures should take no .15 min according to half of the interviewees, while the other half suggested that any .10 min would be problematic: ‘When I was doing Moodgym [an Australian online depression treatment resource] it was 50 000 questions and by the end I said stuff this’; ‘people will get bored after 5 min and . . . they’ll just tick at random’.<br><br>One interviewee suggested dividing questions into sections (e.g. titled ‘positive stuff’ etc.) to make the task less daunting. Three interviewees suggested providing brief personalized feedback about scale scores and graphs showing changes at follow-up assessments.<br><br>One interviewee suggested that participants be asked to log how much practice they had done and how their mood had been each week before viewing videos. He commented that this may provide useful data and encourage participants’ progress in the programme especially if it was followed by an automated message of encouragement.<br><br>All agreed that young people were likely to be eager to provide qualitative feedback about their experience of the programme. They emphasized that the plan to devise a future, improved version of the programme based on their feedback be highlighted at the outset. One interviewee suggested asking	

	‘what are the things that have changed in your life since you began the program?’ upon completion to capture any adverse or stressful life events. Two were concerned that random allocation was likely to deter many potential participants but suggested that a waitlist rather than nonintervention control model would mitigate this effect somewhat.<br><br>**Advertising and recruitment**<br><br>Four interviewees suggested avoiding any reference to ‘spirituality’ or even ‘meditation’ as ‘they might think it’s some spiritual bull . . . !’ They suggested instead focusing on the likely benefits of the programme. Suggested terms were: ‘stress reduction’; ‘managing procrastination’; ‘getting your anxiety down’; ‘stop stressing out’; ‘relieve the stress of everyday life’; ‘relieve the pressure of everyday life’; ‘increase your happiness’; ‘learn to listen to yourself’ and ‘become aware of your reactions’. One interviewee advised caution in using references to stress or ‘stress management’ as in his view potential participants were already (especially in educational settings) ‘bombarded’ with tips on reducing stress. Aside from formal avenues of advertising one interviewee suggested the study have a Facebook and a Twitter presence. One interviewee suggested a video of a young person who has had MT previously speaking about the benefits of the programme on the website homepage.<br><br>**Potential problems envisaged by interviewees**<br><br>Ten interviewees commented that although the idea of learning mindfulness skills is likely to appeal to many young people, persevering in the programme to the end and completing home practices were likely to prove difficult. One interviewee pointed out that young people may be reluctant to undertake	

	the programme because of concerns about privacy (e.g. when using a family or other public computer).<br><br>Description of changes researcher made considering user feedback:<br><br>**An outline of the MATE programme informed by input from young people**<br><br>The brief draft MATE programme originally presented to interviewees was refined and elaborated based on the aforementioned input as noted in the Table 1.<br><br>All suggestions outlined above were incorporated into the design of the website, evaluation plan and advertising activities (though not all are detailed in Table 1 for conciseness). The following were exceptions: omission of references to ‘meditation’ and ‘stress’; phrasing initial registration as an ‘application’; limiting outcome measures to take no longer than 10 min to complete (suggested by half); including functional outcome measures beyond what is captured in the assessment of subjective well-being and personalized feedback regarding scale scores and programme progress. The rationale for exclusion is discussed below.<br><br>From the discussion section:<br><br>Interviewees commented that such a programme was likely to be well received by young people and offered an extensive range of suggestions for improvement centred around the following themes: ‘look and feel’ of the webpage; information delivery medium and pattern; provision of feedback to participants; making homework practical and appealing; volume and composition of outcome measures and qualitative enquiry; tailoring advertising material and avenues to young	

	people and strategies for retaining participants in the programme.<br><br>Formative feedback has been an integral part of programme design in the education setting since the 1960s (Van den Akker et al., 1999). Its use in health promotion has been a later development (Dehar et al., 1993). This is especially so where young people are concerned (McGraw et al., 2000). Empirical evidence has only recently begun to emerge that obtaining and implementing such feedback leads to improvements in programme outcomes (Brown and Kiernan, 2001).<br><br>Nonetheless, in youth-specific research, there is a strong drive to involve young people from the very earliest stages of intervention development (Stafford et al., 2003). This involvement is often within the bounds of an adult agenda, distinguishing it from true youth participation involves young people deciding on the agenda itself (National Children’s Advisory Council, 2009). Consultation, of which this study is an example, entails, by its nature, a balance of power biased towards adults: they determine the questions to be asked and decide what to do with the information (National Children’s Advisory Council, 2009).<br><br>Notably, in a study exploring the views of children and young people about the consultation process, Stafford et al. found that young people saw this form of participation as very valuable (Stafford et al., 2003). They suggested that the process should be: inclusive of a wide variety of young people; motivated by a genuine interest in making use of their views; have a clear purpose that is well communicated to young people and is respectful of young people’s role as making an important contribution (Stafford et al., 2003).	

	We used a variety of methods to reduce the imbalance engendered in a consultation approach: provision of a minimal amount of draft content so as to 'lead' interviewees as little as possible; a minimum number of standardized questions with an open format and allowing flexibility in responding to material provided by interviews by using additional, unscripted clarification questions. We found that face-to-face interviews provided richer and more detailed data than telephone interviews (held with participants geographically distant from researchers). Participants appeared eager to engage in the process and required little encouragement to elaborate. Additionally none reported feeling tired, requiring a break or termination of interview.<br><br>A common challenge in making use of consultee feedback is the fundamental tension between what a health promotion professional or researcher may feel is important to include in a programme (e.g. randomization) and what potential recipients may desire. Negotiating differences may be particularly difficult when initial consultation is held, as in this study, without participants having actually experienced the programme in full and understood its characteristics and rationale experientially (e.g. regarding sustained meditation practice if attentional skills are to be improved). A balance should ideally be struck between respect for the need for autonomy and the self-knowledge that a chosen target population brings and 'expert' knowledge based on previously available evidence of what works and what does not (Castro et al., 2004). Using a blueprint for interviews, in the form of an intentionally brief draft (Table 1), allowed us to 'insist' on some key aspects, which we thought were necessary (e.g. practice between teaching sessions). Also we chose not to follow some suggestions to ensure accurate and ethically sound	

	communication to potential participants and effective programme evaluation: that words such as ‘meditation’ or ‘stress’ be omitted from advertising; that registration is phrased as an application process; and that questionnaires not take .10 min to complete (versus the other half of participants who suggested a maximum of 15 min).<br><br>Some suggestions could not be incorporated into the final programme design as the software costs would have been prohibitive: personalized automated feedback based on outcome measure scores; automated reminders to do ‘timeout’ practice; and online animations. Additionally, some suggestions from participants were in conflict with others, which meant compromises had to be made in incorporating them. For example, minimizing the number of questions asked during outcome measurement was unanimously agreed on. Nevertheless some participants suggested additional items to be added to the already broad range of domains they had felt was relevant.<br><br>An ideal alternative consultation study design would perhaps involve interviews with a group of young people with extensive personal training in mindfulness or as mindfulness educators. Such a group could then be presented with no draft plan at all but simply with the question: ‘How would you design an online MT program for young people?’ This exercise was not undertaken in this study as recruiting a sufficient number of young people with extensive personal experience in this area was not deemed possible. Additionally, interview feedback may be enriched had we circulated a summary of the results back to participants and conducted a second round of interviews. We aim to redress these issues in a detailed qualitative study planned as part of the evaluation of the online	

			MATE programme once it has been finalized and delivered based on the results of this initial consultation.		
**Study**<br><br>• Authors<br>• Publication year<br>• Title	**Country**<br><br>• Conducted<br>• Published	**Study design**<br><br>• Qualitative, mixed methods, or intervention study (type)<br>• Pilot or full-scale<br>• Brief content of question(s) to evaluate user experience	**Aims/purpose**<br><br>• Direct (verbatim) quotations	**Sample**<br><br>• Sample size<br>• Recruitment details<br>• Notable characteristics	**Psychosocial outcomes**<br><br>• Construct(s) measured or targeted by the intervention
• Kubo, A., Aghaee, S., Kurtovich, E. M., Nkemere, L., Quesenberry, C. P., McGinnis, M. A. K., & Avalos, L. A.<br>• 2021<br>• mHealth mindfulness intervention for women with moderate-to-moderately-severe antenatal depressive symptoms: A pilot study within an integrated health care system	• California<br>• California	• Intervention study (single-arm trial), pilot project<br>• Assessed acceptability through responses in a semi-structured interview<br>• "Women were asked open-ended questions about their experience with the study and with Headspace, recommended changes to the study procedures, and perceived need for additional health	• …little is known regarding the efficacy of self-paced, mobile-delivered (mHealth) mindfulness interventions in this population. This study tested the feasibility and acceptability of offering such an intervention for pregnant women with moderate-to-moderately-severe depression symptoms	• N=20 completed (n = 27 enrolled, n = 7 lost to follow-up)<br>• Recruited from obstetrics and gynaecology clinics<br>• 18 years+, score 10-19 on PHQ-9 (moderate-to-moderately-severe depression symptoms), <28 weeks gestation, must not be engaging in regular	• Depression, stress, sleep quality, mindfulness

		system support for pregnant women."<br>• "*Integration* was assessed by asking participants during the semi-structured interview about perceived effects/benefits of practicing mindful-ness and intention to continue using the Headspace program."<br>• Used thematic analysis: "Data from semi-structured interviews were uploaded into NVivo qualitative data analysis software (QSR International Pty Ltd., version 12, 2018.). Inductive thematic analysis was used to identify and develop codes on themes related to mind-fulness benefits, the interface experience,		mindfulness or meditation 3+ times per week<br>• 65% White, 75% college degree<br>• Aged 19–39 years (M = 31)	

<table>
<tr><td></td><td></td><td>experience with the study, and suggested changes. Two primary coders (MM and LN) coded each interview. A third coder (EK) reviewed all coded transcripts to ensure accuracy of codes.”</td><td></td><td></td><td></td></tr>
<tr><td colspan="3">Intervention description

• Type, duration, comparator</td><td colspan="2">User experience

• Any data related to user experience, including methodological considerations (e.g., recruitment strategies, evaluation method, modifications to the intervention)
• Direct (verbatim) quotations</td><td>Other findings reported
• Useful not captured</td></tr>
<tr><td colspan="3">• Participants asked to follow self-paced 6-week mindfulness meditation program using Headspace mobile app
• Practice 10-20 min daily
• Completed 30-day “Basics” course then could choose other 10- to 30-day courses that are condition- or situation-specific
• “All Headspace courses teach mindfulness using various basic techniques, including breathing exercises, body scan, noting (being aware of any emotions that may be arising at the moment), and visualization (visualizing images such as sun shining on the entire body). In addition to the daily, progressive audio instruction, there are occasional short (1–2 min) lecture videos designed to increase the understanding of mindfulness and to encourage its integration into daily life.”</td><td colspan="2">From the results section:

Acceptability

Fifteen participants (75%) completed a brief, post-intervention telephone interview. Ten participants (67%) who responded reported that they were either “very” or “extremely satisfied” with the Headspace app. Overall, participants felt it was easy to use, appreciated the convenience of the app (e.g., able to use at any time and any location), and liked the variety of meditation packs available. Some participants disliked the meditation facilitator’s voice and reported they would have liked to have different voice options.</td><td></td></tr>
</table>

	**Integration**<br><br>The most commonly reported perceived benefits of the meditation program were decreased anxiety, increased calmness, improved sleep, and being able to set aside time for them-selves and to quiet their minds. Most felt that the first trimester was the best time to start the meditation program, preferably as early as possible after learning about the pregnancy. All participants interviewed said they were planning to continue using Headspace after the end of the study.<br><br>From the discussion section:<br><br>Participants appreciated the convenience of the intervention, and many learned mindfulness skills using mHealth technology.<br><br>In the present study, the participants appreciated the intervention because of the ease of using the program and the short dosage (10–20 min a day). They also welcomed the fact that they could use their mobile phone or computer to engage in the intervention without having to leave home or work to attend in-person classes. Most women we reached had access to the technology to receive the mHealth intervention, and among those who enrolled, very few participants had trouble downloading or using the app.<br><br>A few lessons were learned from this feasibility study. First, the recruitment rate was higher when clinicians referred patients to the study compared to when study staff reached out about the study.	

	This underscores the importance of engaging clinicians when conducting intervention studies to maximize recruitment rates. Second, we tracked participants' app usage and reached out when there was an extended period of inactivity (i.e., <3 sessions in the past week). This helped remind some participants to get back on track with the app. Using the reminder features and push notification built into the app may also be useful as a reminder given the proportion of women our study team reached out to with reminder calls. Additionally, automatic tracking of progress can be used as a source of motivation to continue using the program. Such interactive features of a mobile app can increase its adherence and effectiveness, and ease of using the app can facilitate widespread, efficient implementation (Hebden et al. 2012).	

Note. All extracted data in the "Aims/purpose" and "User experience" columns are direct (verbatim) quotations. Quotation marks ("") are used to denote direct (verbatim) quotations in other columns.
