# Supplementary material for: Experiences of Using Digital Mindfulness-Based Interventions: Rapid Scoping Review and Thematic Synthesis
Source: J Med Internet Res. 2023 Sep 28;25:e44220. doi: 10.2196/44220 (PMC10570895; doi:10.2196/44220)
Supplement: Multimedia Appendix 6 [file jmir_v25i1e44220_app6.pdf]

Article title: Experiences of Using Digital Mindfulness-Based Interventions: Rapid Scoping Review and Thematic Synthesis

Journal name: Journal of Medical Internet Research (JMIR)

Author names: Emma L. Osborne, Ben Ainsworth, Nic Hooper, Melissa J. Atkinson

Corresponding author: Emma L. Osborne, Department of Psychology, University of Bath, Claverton Down, Bath, BA2 7AY, UK; Email: elo25@bath.ac.uk

Multimedia Appendix 6: Data Analysis

As recommended in the Cochrane Handbook for Systematic Reviews of Interventions [23], we thematically synthesised extracted data following three stages [38]. Thematic synthesis offers a clear and accessible inductive approach to produce descriptive themes that can evolve beyond the content of the primary studies into more in-depth analytic themes. The first author imported all extracted data verbatim into NVivo software and freely coded the data line-by-line according to its meaning and content, using words directly from the data where possible. For example, one code was “Not training induces feelings of guilt” (see **Table 1** for a four-page excerpt of the 420 codes from 570 references). Considering qualitative evidence synthesis has received criticism for decontextualising the findings of individual studies [38], the first author read all the extracted data (including study aims, methods, and sample) prior to coding each study’s findings to preserve its original context and ensure its findings could be fully understood without misinterpretation [39]. The first author then grouped similar codes into “descriptive themes” to summarise their meaning while keeping close to the original findings of the included studies. This was an iterative process that produced a total of 15 descriptive themes which distilled users’ perspectives and experiences of using digital MBIs down into their key parts (e.g., “Not feeling part of a community”). **Table 2** provides a full list of descriptive themes.

In the next stage, the wider research team met to discuss the descriptive themes and develop “analytical themes”, which go beyond the findings of the primary studies by interpreting the key messages underlying the descriptive themes and using these to answer the review questions. We generated more abstract and analytical themes through an iterative process of inferring barriers, facilitators, and implications for intervention development from the descriptive themes, and making changes to these where necessary. For example, four descriptive themes related to a dependence on others (essential role of a “support person”, involving significant others, supportive communication, not feeling part of a community). From these, we inferred several barriers and facilitators. Participants desired a “community component” so that they could overcome challenges and discuss their experiences with other users. Participants also felt it was reassuring to know someone was available if needed to clarify content and provide technical or administrative support. These perspectives indicated that people engaging in digital MBIs often depend on someone or something else (e.g., a therapist, researcher, significant other, another participant, or the programme itself) for support and encouragement. We captured this in the analytical theme entitled “Leaning on others”. Altogether, this process produced three analytical themes (Making Mindfulness a Habit, Responses to Own Practice, and Leaning on Others).

Table 1

Excerpt from our thematic synthesis

Code label	Coded data
Not training induces guilt feelings	Experiencing the training as another stressful demand, not training induces guilt feelings
Berated self when did not practice but then was gentle and tolerant	MS. G: There was a lot more homework this week and the body scan practice took a long time. I have perfectionistic tendencies, like I want to try to do all the practices every day just the way I am supposed to. The other night, I fell asleep during the body scan practice. I'm just so exhausted in this pregnancy! At some point I became alert and realized I had fallen asleep. At first I kind of berated myself, but then I was like "you had a long day, and you're pregnant! It's okay." I kept following along with the recording where it was.
Felt guilty, self-critical, resentful toward time demands	Additionally, some believed that the expectation for time commitment was unreasonable and felt guilty or resentful that they could not meet it. For example, in response to a question about her experience with home practice, this participant describes the self-criticism she experienced as well as some benefit from the practice: "[I felt] a little critical of self, felt like I couldn't do it all, and it was my fault somehow, and this is too much to ask with your daily life, and resentful. But I tried my best to do it all, gave it a pretty good effort, and got stuff out of it."
Trying to find time to fit in practice increased rather than reduced their stress	Participants from nearly 50% of call centers in the intervention reported not having a designated time-off to complete the intervention despite the intervention protocol. This was a major barrier for completing the intervention content, and some participants believed the intervention increased rather than reduced their stress. For example: I am finding it is almost causing more stress trying to find the time to get practice in and to do the weekly lessons. We do not have the staffing to permit us time off the floor to complete training, so we must do it while on duty on the floor.
Felt angry at self when no time for meditation	With project deadlines in parallel it is hard to choose a time for meditation, very angry at myself
Felt guilty when missed a day due to other commitments	I did miss a day. I missed a Saturday as I had to do some work at a nonprofit organization and we didn't get done until late. I totally forgot about it. I felt guilty about it, but sometimes that happens. But because I feel frequently overwhelmed like I can't quiet my mind, I've been listening to the meditations more regularly now, especially at night.
Demands of training led to stress	Some negative effects of training were reported, e.g., stress or distressing feelings in connection with the training.

Feel guilty when practise as not enough time in day to be resting	Focus group participants expressed a few reasons why they may have difficulty participating in some of the exercises, including physical limitations, time commitments, and feelings of guilt. For example, a couple of participants felt that they may not take the time to complete the exercises because they were busy. One woman also said, I feel guilty when I do stuff like this…because it's like there's not enough time in the day to be resting."
Takes time to create a routine	It takes time to create a routine
Responsibility for own time management as a barrier	Responsibility for your own time management was mentioned as a barrier because it required a lot of self-discipline
Requires discipline	It's a question of discipline /. . ./ I think one should pinpoint that it's strenuous and that one has to be ready to struggle with it because one believes in it.
Requires discipline	The mindfulness training was seen as a valuable tool to handle stress and make time for a daily break, but it required discipline.
Requires discipline	Responsibility for your own time management was mentioned as a barrier because it required a lot of self-discipline. One patient stated: What I like about it is that I can manage my own time which went very well the first couple of weeks. After a while some chores interrupted me and then at the end of the day I realized: I still have to practice. Sometimes I did not do it anymore and sometimes I did. So you have to be very disciplined to stick to the schedule.
Recommended drawing a parallel between home practice and ritual/regularity of medication	Another suggested drawing a parallel with the ritual and regularity of 'when you're on a medication' when describing the approach to practice.
Made a commitment to integrate into life	An aspect of this commitment to continue practicing mindfulness and acceptance was an inner resolution to integrate this practice into their lives: You just have to make time for it like you make time for anything else you want to do. You just have to work for it if this is something that you want. Instead of it being like a burden like, "Oh, I have to do this mindfulness exercise." You have to really want it to be able to be better, so then you do.
Had to adjust schedules and obligations to make mindfulness a habit	As the participants began to accommodate the daily use of the app into their already busy personal, academic, and professional schedules, they encountered the challenges of establishing a new habit. For the participants, this was not a straightforward process, but rather involved several adjustments in their schedules, priorities, obligations, and struggles with their posttraumatic stress symptoms.
Encourage practice at same time every day to make routine activity	To make home practice engagement more likely three interviewees suggested asking participants to practice at the same time every day perhaps 'pegging it' to a routine activity (e.g. after brushing their teeth in the morning).

Creating a routine was experienced as a challenge	Motivation and barriers to training were mentioned. Primarily, creating a routine was experienced as a challenge that required an active decision and prioritising.
Creating a daily training routine was easy for some and difficult for others	Creating a daily training routine came through as easy for some participants and difficult for others. This partly depended on one's general life situation, with potentially stressful life events, and other competing commitments.
Establishing daily training routine facilitated adherence	Being successful with establishing daily training routines facilitated adherence to the recommended dose, while still allowing flexibility to skip or redo a meditation exercise depending on the circumstances and prerequisites to do the training.
Had to remind self at beginning but then became routine	In the beginning, I had to remind myself. But now, now it has become more like a routine.
Reminders were helpful without being intrusive	A consistent message from all interviewees was that any form of feedback or communication from the programme was likely to improve retention. In addition to forms of feedback already mentioned, email (even if automated and using a 'no-reply' address), and text message reminders, were thought to be likely to be helpful without being intrusive.
E-mail reminders were encouraging	E-mail reminders were experienced as encouraging reminders by a majority of participants.
Positive or neutral stance towards reminders	Most participants had a positive or neutral stance towards the weekly/biweekly reminders.
Liked reminders	There was strong endorsement for the live administrative support and reminders in MMB [Mindful Mood Balance intervention].
Wanted automated messages of encouragement	One interviewee suggested that participants be asked to log how much practice they had done and how their mood had been each week before viewing videos. He commented that this may provide useful data and encourage participants' progress in the programme especially if it was followed by an automated message of encouragement.
Reminders to were enjoyable and helped participants remember to practice	The app was super easy to navigate. It was completely 100% userfriendly. It was very functional. I enjoyed the reminders that the app sends you – I really found that helpful because otherwise, I would not have remembered to do it.
Would like regular contact from support person asking 'how are you'	Just two three times a week a brief moment of contact saying "how are you"?
Would like live administrative support	There was strong endorsement for the live administrative support and reminders in MMB. While participants were informed of the home practice time commitment during the consent process, perhaps there is a misconception that an online program implies a lesser time commitment. It will be important for future iterations of MMB to refine the support function, potentially by adding an online community,

	and also to clarify the importance and expectations for home practice at the outset.
Valued support person to clarify practical aspects that were unclear	Patients indicated that the therapist was often able to clarify practical aspects that were unclear.
Valued support person to clarify practical aspects that were unclear	The ability to express themselves in writing was very helpful for some to give words to their subjective experiences and to ask for clarification to the therapist if it was necessary.
Most valued the support of a coach	The majority reported that they valued the support of a coach
Therapist interaction made participant feel connected	(My therapist) was very patient and gave me all the space I needed [...]. She was like this all the time, in everything she did, not forcing, but stimulating me. “Do it for yourself when you do the exercises. If you do them, you could benefit a lot.” This made me feel more connected.
Wanted more support for troubleshooting session content	Adding increased support was suggested for troubleshooting session content as well as managing time challenges for completing home mindfulness practice.
Nice to have someone available to contact	The benefit of reminders and follow-up contacts also was mentioned: “I think it’s nice to have someone available, whether someone wants it or not. It was nice that you were like, ‘you haven’t logged on in a while’…it’s hard to stay on track.”
Lack of direct contact with instructors reduced accountability for completing sessions	A noted limitation of the Web-based program was the loss of direct contact with instructors compared to in-person groups and the reduced accountability for completing sessions and home practice.
Liked the 'Ask a Question' function	Others described a desire for feedback from a professional therapist in person and some suggested professional feedback could be provided to reflection question inputs. Some saw the “Ask a Question” function as a good way to have some interaction beyond the website and enjoyed posting or just reading materials in this section posted by other users. Suggestions were provided for adding a community component such as: Web-based message boards with other users, hold a few in person meetings (although the logistical scheduling barriers to this approach were recognized), or create opportunities for telephone and/or video chat meetings.
Human support and feedback to facilitate engagement	Our results revealed that the ACT-based app users might require human support and feedback during the intervention period.
Human support and feedback to facilitate engagement	Furthermore, our results also showed that mental health app developers should carefully consider integrating processes that enhance app user engagement, such as those providing human support and feedback (i.e., emotional and technical support).
Found support person essential	Expressed a belief that having a program support person was an essential part of program
Found support person essential	Of those who completed the program, however, many endorsed that it was “essential” to have a coach and helpful to know that one was available if needed.

Table 2

Descriptive themes and details from the study findings

**Barriers**	
**Descriptive themes**	**Details from the study findings**
Scheduling time to practice may be challenging	• Some participants experienced difficulties with discipline, which they viewed as essential for managing their time, adjusting their priorities and obligations, and establishing a new habit.<br>• Some participants reported that it took time to create a training routine but achieving this made completing the program easier.<br>• Some participants felt that they did not receive necessary guidance on how to incorporate the program into their lives and make practice a scheduled activity.<br>• Some participants reported that there was just not enough time to fit practice into daily life.<br>• Some participants found it difficult to remember to practice, especially in the busyness of daily life.<br>• Some participants felt guilty, resentful, and self-critical when they could not find time to practice or meet the expected time commitment, leading them to experience practice as another stressful demand.
Physical and psychological distractions impeding engagement	• Some participants reported physical distractions that prevented them from engaging in practice, including frequent interruptions, noise levels, and shared spaces.<br>• Some participants experienced difficulties controlling their attention because they regularly found themselves distracted by their thoughts (e.g., daily plans, negative emotions, life problems).<br>• Some participants felt frustrated by these disturbances.
Negative responses to certain concepts	• Some participants felt hesitation and scepticism towards mindfulness, and they considered certain aspects ‘dopey’ or ‘bluff’.<br>• Some participants perceived mindfulness as a now ‘fashionable trend in society’, but also that it was timely.<br>• Some participants felt the language used was abstract or ‘foreign’ to them (e.g., compassion training, specific figures of speech).<br>• Some participants disliked the term “homework” because it reminded them of university/school work or rendered practice a stressful ‘responsibility’ they must fulfil each day.<br>• Some participants reported that reference to “spirituality” or “meditation” carried a religious overtone, leading them to feel doubtful and suspicious about the intervention’s credibility.

Lack of understanding and "doing it right"	• Some participants felt unsure about what was expected of them or what the program required (e.g., whether they could manage it, what they should prioritise, what should happen during practice).<br>• Some participants experienced difficulties understanding the instructions for daily meditations, the purpose of individual practices or the overall program, and the rationale for mindfulness.<br>• Some participants were surprised by the expected time commitment despite information provided during consent, indicating a potential misconception that an online program implies a less time.<br>• Some participants had a sense of insecurity about whether they were practising properly. They questioned the accuracy of their training (e.g., when they fell asleep, whether brief practices "count" or they had "permission" to do a briefer practice when short of time, whether they were in the correct position or "doing it right").<br>• Some participants felt puzzled about the effects they were experiencing after practising mindfulness.
Concerns over privacy	• Some participants were concerned about the privacy of their information, safety of the website, and anonymity of the platform. This was particularly true of younger participants (e.g., when using a family, public, or other shared computer).
Difficult when "needed it the most"	• Some participants found it difficult to practise when they felt they needed it most, i.e., when stress and anxiety levels were higher, when they had no spare time.
Not feeling part of a community	• Some participants desired a community component (e.g., online forum, group message board) so they could discuss their intervention experiences, clarify content, and share/overcome challenges, with other users. This was particularly wanted by participants with a shared lived experience so they could interact, connect, and identify with other users (e.g., perinatal women, people with epilepsy, cancer patients).<br>• Some participants felt alone or that they lacked connection and a sense of belonging with other users.
Contradictory preferences: "Different things work for different people"	• Variation in sound preferences: Some participants reported there was too much narration during the practices, and they found the voice irritating, distracting, or disturbing. They wished for more silent periods to allow time to settle and meditate. Some participants appreciated the narrators' voice and felt it was agreeable, easy to follow, and provided helpful guidance. Some participants did not like the specific sound of the voice (e.g., found it sad, not soothing or calming) or reported they would have liked to choose between different voice options. Some participants preferred to practice in a quiet or silent environment, whereas others found background music or ambient sounds (e.g., of nature) made practice easier.<br>• Variation in time preferences: In addition to the majority preference for shorter practices described above, some participants preferred longer practices because they allowed time for the mind to slow down and for

	participants to concentrate better. Some participants experienced the same practices as being too long and other participants as too short.<br>• Variation in variation preferences: Some participants viewed repetition in practice content as essential, familiar, and safe, whereas others thought this was monotonous, distracting, and wished for more variation.<br>• Variation in reminder preferences: Some participants viewed reminders as helpful whereas others viewed them as condescending.
**Facilitators**	
**Descriptive themes**	**Details from the study findings**
Helpful role of a "support person"	• Some participants valued the support of an individual (e.g., instructor, therapist, member of the research team) with whom they could discuss program concepts and receive technical/administrative support.<br>• Some participants felt it was reassuring to know someone was available if needed, whether via phone, email, or an "Ask a Question" / "Help" function.
Supportive communication	• Some participants reported that any form of communication from the program (e.g., automated reminders, messages of encouragement, personalised feedback, via email or text) was helpful in reminding and motivating them to practice without feeling intrusive. However, (multiple) daily check-ins were disliked.
Preferred series of short practices	• Some participants preferred multiple, short practices during each week rather than one long session because shorter practices are more attainable with respect to remaining attentive (i.e., minimise interruptions and loss of focus), scheduling (i.e., easier to make time for and integrate into daily life), and avoiding adverse experiences (i.e., boredom, impatience, and discomfort from sitting still).
Benefits perceived as motivating	• Some participants experienced benefits from consistent engagement (e.g., improved sleep, calmness, and managing of stress and worry), which made them feel encouraged and more willing to continue. Conversely, lack of awareness of the positive changes initiated by regular practice prompted disengagement.<br>• Some participants reported that their participation in a research study provided an opportunity to benefit others and be part of a wider project with moral goals, which motivated them to remain involved.<br>• Some participants reported physical rewards would motivate them to engage (e.g., certificate of completion, monetary prize draws with a higher chance to win small prizes).
Appreciated ease and flexibility of use	• Some participants appreciated the ease of using the intervention, including its accessibility, readability, and simplicity with respect to layout, structure, and guidance. Conversely, technical difficulties (e.g., navigation problems, crowded design, too many clicks, complicated instructions) presented a barrier to use.

	• Some participants valued the convenience of the intervention in that you could use it at any time/location and on various personal devices, including mobile phone, tablet, and computer.
Helpful downloads	• Some participants reported that being able to download session materials (e.g., as PDF files, in MP3 or MP4 format) would be helpful in reviewing content and supporting practice by providing a daily reminder.
Involving significant others	• Some participants reported sharing the program with significant others and doing practices together. They felt that supportive friends and family reinforced their consistent and continued practice.<br>• Some participants appreciated help from significant others in “protecting time” for longer practices.
