# Supplementary material for: Experiences of Using Digital Mindfulness-Based Interventions: Rapid Scoping Review and Thematic Synthesis
Source: J Med Internet Res. 2023 Sep 28;25:e44220. doi: 10.2196/44220 (PMC10570895; doi:10.2196/44220)
Supplement: Multimedia Appendix 7 [file jmir_v25i1e44220_app7.pdf]

Article title: Experiences of Using Digital Mindfulness-Based Interventions: Rapid Scoping Review and Thematic Synthesis

Journal name: Journal of Medical Internet Research (JMIR)

Author names: Emma L. Osborne, Ben Ainsworth, Nic Hooper, Melissa J. Atkinson

Corresponding author: Emma L. Osborne, Department of Psychology, University of Bath, Claverton Down, Bath, BA2 7AY, UK; Email: elo25@bath.ac.uk

Multimedia Appendix 7: Methodological Streamlining

We took several steps to accelerate the review process so that evidence could be quickly incorporated into the early stages of intervention planning [40]. We transparently report on these here so that readers and decision makers are aware of the potential trade-off in validity and can make their own decision about the effect of potential biases. Nonetheless, results from studies comparing rapid reviews and full systematic reviews suggest they arrive at similar conclusions [43,44].

First, we limited inclusion criteria to English-language publications as recommended by the Cochrane Rapid Reviews Methods Group [25]. Evidence suggests that exclusion of non-English publications has a minimal effect on conclusions from systematic reviews of interventions and may therefore be a practical methodological shortcut [45].

Second, we restricted searching to PsycInfo because: (a) too much data due to the inclusion of many studies can weaken qualitative syntheses (e.g., by preventing a detailed engagement with text), (b) there is currently no consensus on how best to limit the number of included studies in a qualitative evidence synthesis and methods used are often time and resource intensive (e.g., purposive sampling) or involve limiting the scope of the research question (e.g., by population), (c) searching a specialist database for psychological interventions [41] to retrieve studies most suitable for answering our objectives is an efficient

way of achieving a manageable amount of relevant data, (d) qualitative evidence syntheses aim to understand the phenomenon of interest in a context rather than aggregate data from large representative samples of studies to achieve statistical generalisability [42], and (e) we identified no substantially new codes or themes towards the end of the thematic synthesis, indicating we had reached conceptual saturation [38].

Third, one reviewer performed full screening and data extraction. We minimised the potential for increased errors and lower reproducibility by: (a) piloting forms, (b) starting full screening only after achieving substantial agreement between two researchers on a random selection of 50 (10% of records) titles and abstracts (see the **Interrater Reliability** section below for details), and (c) discussing uncertainties and verifying included articles with the research team. Using a single reviewer to extract data in a piloted form ensured data from all studies were extracted in a harmonised way (e.g., consistency in format and level of detail).

Interrater Reliability

We calculated Cohen's kappa (κ) – a measure of chance-corrected agreement [46] – to test interrater reliability. We evaluated the level of agreement between the two reviewers' judgement on whether the subsample of 50 records were eligible or ineligible according to the inclusion criteria. Initially, there was substantial agreement between the two reviewers' judgements, $\kappa = .703$ (95% CI, .433 to .973), $p < .001$. The reviewers achieved 100% agreement after discussing each discrepancy.
