# Supplementary material for: Experiences of Using Digital Mindfulness-Based Interventions: Rapid Scoping Review and Thematic Synthesis
Source: J Med Internet Res. 2023 Sep 28;25:e44220. doi: 10.2196/44220 (PMC10570895; doi:10.2196/44220)
Supplement: Multimedia Appendix 8 [file jmir_v25i1e44220_app8.pdf]

Article title: Experiences of Using Digital Mindfulness-Based Interventions: Rapid Scoping Review and Thematic Synthesis

Journal name: Journal of Medical Internet Research (JMIR)

Author names: Emma L. Osborne, Ben Ainsworth, Nic Hooper, Melissa J. Atkinson

Corresponding author: Emma L. Osborne, Department of Psychology, University of Bath, Claverton Down, Bath, BA2 7AY, UK; Email: elo25@bath.ac.uk

Multimedia Appendix 8: Study Selection

We identified 510 unique records from the database search on 13 September 2021. Of these, 79 entered full-text screening. We contacted the authors of four papers to request missing information (e.g., the sample age range where only *M* and *SD* were reported) or additional details for clarification (e.g., to confirm the intervention was delivered online). One author replied and provided information to confirm ineligibility. We were not able to contact the remaining three authors and so assessed eligibility using available information (see below for our justifications). We ran a top-up search on 30 November 2021 to identify articles published since the original search. The top-up search was performed exactly as it had been run on 13 September 2021 and identified 20 unique records. Of these, 3 entered full-text screening. After repeating the comprehensive screening process, we did not include any additional articles. We included a total of 22 articles in the review. The selection process is described in the adapted PRISMA flowchart (Figure 1 [49]).

Four papers did not contain sufficient information for us to assess eligibility. Three did not report the sample age range (i.e., reported *M* and *SD* only [48,50,51]) and one did not make it clear that the intervention was delivered online [47]. We contacted the authors of these four papers to request missing information or additional details for clarification, as appropriate. One author replied to confirm that the age range of the sample was 40–71 [51],

which confirmed that the study did not meet our inclusion criteria (i.e., the age range must include people between 16–35 years). The remaining authors did not respond to our request, and we therefore assessed eligibility using the available information.

We included the study by Price-Blackshear et al. [48] because the mean age of the sample was 39.67 (*SD* = 5.44, *n* = 36) and 38.78 (*SD* = 5.08, *n* = 41) in the two conditions. As the *M* - *SD* < 35 in both conditions, the sample must include participants aged < 35 years, which satisfies our inclusion criteria. We also included the study by Monshat et al. [47] because the methods stated that “Characteristics and development of the MT [mindfulness training] program employed and its initial evaluation are detailed elsewhere (Monshat, 2011, in peer review)” (p. 573), and the MT programme described in this earlier paper is online [35]. However, we did not include the study by Kubo et al. [50] because the mean age of the sample was 65.8 (*SD* = 8.8, *n* = 52) and 60.7 (*SD* = 16.5, *n* = 22) in the two conditions, which was substantially higher than the age range specified in our criteria and therefore unlikely to include people between 16–35 years.
