# Supplementary material for: Experiences of Using Digital Mindfulness-Based Interventions: Rapid Scoping Review and Thematic Synthesis
Source: J Med Internet Res. 2023 Sep 28;25:e44220. doi: 10.2196/44220 (PMC10570895; doi:10.2196/44220)
Supplement: Multimedia Appendix 9 [file jmir_v25i1e44220_app9.pdf]

Article title: Experiences of Using Digital Mindfulness-Based Interventions: Rapid Scoping Review and Thematic Synthesis

Journal name: Journal of Medical Internet Research (JMIR)

Author names: Emma L. Osborne, Ben Ainsworth, Nic Hooper, Melissa J. Atkinson

Corresponding author: Emma L. Osborne, Department of Psychology, University of Bath, Claverton Down, Bath, BA2 7AY, UK; Email: elo25@bath.ac.uk

Multimedia Appendix 9: Study Characteristics

Table 1

Study characteristics.

**Variable**	***n***
Year published	
2010	1
2011	1
2013	1
2014	1
2016	1
2017	6
2018	1
2019	1
2020	3
2021	3
2022	3
Corresponding author country	
USA	11
Europe (Sweden, Italy, Netherlands, Russia)	6
Australia	4
China	1
