# Supplementary material for: Experiences of Using Digital Mindfulness-Based Interventions: Rapid Scoping Review and Thematic Synthesis
Source: J Med Internet Res. 2023 Sep 28;25:e44220. doi: 10.2196/44220 (PMC10570895; doi:10.2196/44220)
Supplement: Multimedia Appendix 10 [file jmir_v25i1e44220_app10.pdf]

Article title: Experiences of Using Digital Mindfulness-Based Interventions: Rapid Scoping Review and Thematic Synthesis

Journal name: Journal of Medical Internet Research (JMIR)

Author names: Emma L. Osborne, Ben Ainsworth, Nic Hooper, Melissa J. Atkinson

Corresponding author: Emma L. Osborne, Department of Psychology, University of Bath, Claverton Down, Bath, BA2 7AY, UK; Email: elo25@bath.ac.uk

Multimedia Appendix 10: Critical Appraisal

Table 1

Critical appraisal of included studies.

Author(s) and year of publication	Question and study design	Selection of participants	Method of data collection	Method of data analysis
Berg & Perich, 2021[a] [52]	Yes	Yes	Yes	Yes
Berg & Perich, 2021[b] [52]	Yes	Yes	Yes	No
Boggs et al. 2014 [53]	Yes	Yes	Yes	Yes
Chittaro et al. 2016 [54]	Yes	Yes	Yes	Yes
Compen et al. 2017 [55]	Yes	Yes	Yes	Yes
Felder et al. 2017[a] [56]	Yes	Yes	Yes	Yes
Felder et al. 2017[b] [56]	Yes	Yes	Yes	Yes
Forbes et al. 2018[a] [16]	Yes	Yes	Yes	Yes
Forbes et al. 2018[b] [16]	Yes	Yes	Yes	No
Kennett et al. 2021[a] [57]	Yes	Yes	Yes	Yes
Kennett et al. 2021[b] [57]	Yes	Yes	Yes	No
Kerr et al. 2019[a] [58]	Yes	Yes	Yes	Yes
Kerr et al. 2019[b] [58]	Yes	Yes	Yes	Yes
Kubo et al. 2021[a] [34]	Yes	Yes	Yes	Yes
Kubo et al. 2021[b] [34]	Yes	Yes	Yes	Yes
Levin et al. 2017[a] [59]	Yes	Yes	Yes	Yes
Levin et al. 2017[b] [59]	Yes	Yes	Yes	No
Monshat et al. 2012 [35]	Yes	Yes	Yes	Yes
Monshat et al. 2013 [47]	Yes	Yes	Yes	Yes
Osin & Turilina 2021[a] [60]	Yes	Yes	Yes	Yes
Osin & Turilina 2021[b] [60]	Yes	Yes	Yes	Yes
Price-Blackshear et al. 2020 (USA)[a] [48]	Yes	Yes	Yes	Yes

Price-Blackshear et al. 2020[b] [48]	Yes	Yes	No	No
Reyes 2021 [61]	Yes	Yes	Yes	Yes
Reyes et al. 2020 [62]	Yes	Yes	Yes	Yes
Stjernswärd & Hansson 2017[a] [63]	Yes	Yes	Yes	Yes
Stjernswärd & Hansson 2017[b] [63]	Yes	Yes	Yes	No
Stjernswärd & Hansson 2017[a] [64]	Yes	Yes	Yes	Yes
Stjernswärd & Hansson 2017[b] [64]	Yes	Yes	Yes	Yes
Stjernswärd & Hansson 2020 [65]	Yes	Yes	Yes	Yes
Trub & Starks 2017[a] [66]	Yes	Yes	Yes	Yes
Trub & Starks 2017[b] [66]	Yes	Yes	Yes	No
Walker et al. 2010[a] [67]	Yes	Yes	Yes	Yes
Walker et al. 2010[b] [67]	Yes	Yes	Yes	Yes
Yu et al. 2020[a] [68]	Yes	Yes	Yes	Yes
Yu et al. 2020[b] [68]	Yes	Yes	Yes	Yes

[a]Assessment of study overall (i.e., as a whole).
[b]Assessment of study with respect to data included in the qualitative evidence synthesis only.
